# Data‑driven cognitive composites predict Aβ‑related decline and progression to MCI: the ADOPIC study

**DOI:** 10.1002/alz.71917

**Published:** 2026-10-11

**Authors:** Bhargav Tallapragada, Brook Galna, Rosita Shishegar, Shaun J. Markovic, Belinda M. Brown, Vincent Dore, Simon M. Laws, Tenielle Porter, Samantha Burnham, Yen Ying Lim, Jason Hassenstab, Christopher C. Rowe, Liang Jin, Jurgen Fripp, James D. Doecke, Duygu Tosun, Michael Weiner, Ralph N. Martins, John C. Morris, Colin L. Masters, Paul Maruff, Hamid R. Sohrabi, of ADOPIC study

**Affiliations:** ^1^ Centre for Healthy Ageing, Health Futures Institute Murdoch University Murdoch Western Australia Australia; ^2^ School of Psychology Murdoch University Murdoch Western Australia Australia; ^3^ School of Allied Health (Exercise Science) Murdoch University Murdoch Western Australia Australia; ^4^ Personalised Medicine Centre, Health Futures Institute Murdoch University Murdoch Western Australia Australia; ^5^ The Australian e‐Health Research Centre, CSIRO Melbourne Australia; ^6^ School of Computing Technologies RMIT University Melbourne Victoria Australia; ^7^ Discipline of Psychology, Counselling and Criminology Edith Cowan University Joondalup Western Australia Australia; ^8^ School of Medical and Health Sciences Edith Cowan University Joondalup Western Australia Australia; ^9^ Alzheimer's Research Australia, Sarich Neuroscience Research Institute Nedlands Western Australia Australia; ^10^ Centre for Precision Health Edith Cowan University Joondalup Western Australia Australia; ^11^ Collaborative Genomics and Translation Group School of Medical and Health Sciences Edith Cowan University Joondalup Western Australia Australia; ^12^ Curtin Medical School Curtin University Bentley Western Australia Australia; ^13^ School of Psychological Sciences and Turner Institute for Brain and Mental Health Monash University Melbourne Australia; ^14^ Institute of Clinical and Translational Sciences Washington University School of Medicine St Louis Missouri USA; ^15^ Department of Medicine Austin Health Heidelberg Victoria Australia; ^16^ Florey Institute of Neuroscience and Mental Health The University of Melbourne Parkville Victoria Australia; ^17^ The Australian e‐Health Research Centre, CSIRO Brisbane Australia; ^18^ Center for Imaging of Neurodegenerative Diseases University of California‐San Francisco San Francisco California USA; ^19^ Departments of Radiology and Biomedical Imaging, Medicine, Psychiatry, and Neurology University of California San Francisco California USA; ^20^ Macquarie Medical School Macquarie University Sydney New South Wales Australia; ^21^ Faculty of Medicine The University of Western Australia Perth Western Australia Australia; ^22^ Washington University School of Medicine St. Louis Missouri USA; ^23^ Cogstate Ltd Melbourne Victoria Australia

**Keywords:** cognitive composite score, cognitive decline, mild cognitive impairment, Preclinical Alzheimer's Cognitive Composite (PACC), preclinical AD

## Abstract

**INTRODUCTION:**

Cognitive composites offer greater sensitivity to amyloid beta (Aβ)‐related decline compared to individual tests for tracking longitudinal cognitive change. We developed and validated data‐driven composites from the harmonized Alzheimer's Dementia Onset and Progression in International Cohorts (ADOPIC) study, addressing gaps in psychometric reporting and multi‐cohort validation.

**METHODS:**

The study included 1964 cognitively unimpaired (CU) participants at baseline (longitudinal subset = 1110). Principal component analysis‐derived domain‐specific and general cognitive composites were evaluated for psychometric properties, sensitivity to Aβ‐related decline, and mild cognitive impairment (MCI) progression prediction compared to the Alzheimer's Disease Cooperative Study – Preclinical Alzheimer's Cognitive Composite (ADCS‐PACC).

**RESULTS:**

Proposed composites demonstrated good to excellent psychometric properties and sensitivity to differentiating CU Aβ trajectories (p < 0.001). General cognition demonstrated longitudinal sensitivity comparable to the ADCS‐PACC (d = −0.20) and equivalent classification performance (area under the receiver operating characteristic curve = 0.70). All composites significantly predicted MCI progression (p < 0.001).

**DISCUSSION:**

The composites perform comparably to the ADCS‐PACC, developed for CU with Aβ, in longitudinal sensitivity while providing comprehensive psychometric characterization and domain‐specific measurement, supporting their utility as outcome measures in preclinical Alzheimer's disease studies.

## Highlights

- PCA‐derived cognitive composites are aligned with previous literature.
- All composites showed good psychometric properties and structural validity.
- Proposed composites showed high sensitivity to Aβ‐related decline in older adults.
- A replicable framework for data‐driven composite development is provided.

## 1 BACKGROUND

Neuropsychological assessment of community‐dwelling cognitively unimpaired (CU) older adults with pathological levels of amyloid beta (Aβ) is crucial in establishing the current level of cognitive function, monitoring the risk of future cognitive impairment, and optimizing clinical trial enrichment.^1^, ^2^ However, cognitive assessment in older adults presents two key challenges. First, a large proportion of CU older adults score at the higher end of possible scores on at least one test within a given neuropsychological test battery, leading to ceiling effects, which reduce the sensitivity of the measures to subtle cognitive changes.^1^ Second, while using a comprehensive neuropsychological battery is ideal, increasing the number of tests increases the risk of a false positive, that is, identifying cognitive impairment in its absence (Type 1 error).^3^ To address these challenges, scores from individual cognitive measures are combined to produce a single score, termed the cognitive composite.

Cognitive composites have shown improved sensitivity in detecting Aβ‐related cognitive decline (defined as the longitudinal cognitive decline associated with the presence of brain Aβ in otherwise CU older adults)^4^ and reduced error rates compared with individual cognitive tests in the context of sensitivity and reliability for tracking longitudinal cognitive change.^5^, ^6^ In CU older adults, various cognitive composites have been developed and applied across different research contexts, including observational studies^7^ and clinical trials.^3^, ^8^, ^9^ Among the various cognitive composites, the Alzheimer's Disease Cooperative Study – Preclinical Alzheimer's Cognitive Composite (ADCS‐PACC) is one of the most widely used measures in CU older adults with evidence of AD pathology.^10^ However, some of the tests included in the ADCS‐PACC, such as the Logical Memory Delayed Recall, have shown practice effects on repeated assessments, while Mini‐Mental State Examination (MMSE) is prone to ceiling effects in CU populations. Despite this, the ADCS‐PACC, or approximations of it, have been applied successfully in previous studies showing sensitivity to Aβ‐related cognitive decline.^11^, ^12^, ^13^, ^14^ Data‐driven approaches may be used to optimize cognitive composite test selection and potentially improve sensitivity relative to theory‐driven approaches.^9^ Specifically, such methods as principal component analysis (PCA) may enhance the sensitivity of composite scores derived from a set of cognitive tests by empirically identifying the tests that capture the most cognitive variance while reducing redundancy from highly correlated measures.

**RESEARCH IN CONTEXT**

1. **Systematic review**: Sensitive and psychometrically robust neuropsychological measures are critical for detecting subtle cognitive changes in preclinical AD. While data‐driven approaches improve the sensitivity of these measures to Aβ‐related decline, their psychometric properties and validation across multi‐cohort studies remain limited.
2. **Interpretation**: Using PCA on harmonized cognitive data from three prospective AD cohorts of the ADOPIC study (ADNI, AIBL, OASIS), we developed four cognitive composites that (a) demonstrate sensitivity to Aβ‐related cognitive decline over 8 years and predict progression to MCI comparable to ADCS‐PACC and (b) offer empirically derived test selection, explicit evidence of structural validity, psychometric characterization across multiple domains, and good performance across harmonized multi‐cohort data supporting generalizability.
3. **Future directions**: Key questions include (a) generalizability to diverse populations, (b) whether domain‐specific trajectories map onto brain regions specific to early Aβ pathology, and (c) whether digital or remote administration of these outcome measures is feasible.

PCA offers several advantages over theory‐driven approaches in developing composites. First, PCA identifies linear combinations of tests that capture the most variance in cognitive performance within a sample, which can be labeled to reflect cognitive domains or clinical features by examining test loadings.^15^, ^16^ Using this approach, significant loading tests may be combined to form a single score to assess cognitive performance within or across cognitive domains.^17^ However, it remains unknown whether optimization of cognitive composites through PCA, applied to large‐scale, multi‐cohort harmonized data, can improve sensitivity to Aβ‐related decline and psychometric validation compared to a theory‐driven composite such as the ADCS‐PACC.^10^

Therefore, in this study, a set of empirical cognitive composites for CU older adults was developed and validated from a large‐scale multi‐cohort international consortium, the Alzheimer's Dementia Onset and Progression in International Cohorts (ADOPIC). The aims of this study were to (1) develop general and domain‐specific cognitive composites using PCA to reduce non‐informative variance; (2) validate the developed cognitive composites by assessing their longitudinal sensitivity to baseline Aβ‐related cognitive decline, psychometric properties, and ability to predict progression to mild cognitive impairment (MCI); and (3) compare the sensitivity of the new composites to Aβ‐related decline against the ADCS‐PACC and MMSE. The main hypothesis was that the new domain‐specific and general composites would demonstrate greater sensitivity to baseline Aβ‐related cognitive decline and better predict progression to MCI than the ADCS‐PACC.

## 2 METHODS

### 2.1 Study data and participants

Study data were obtained from the ADOPIC study, which harmonizes cognitive, Aβ‐positron emission tomography (PET) imaging, and demographic data across three large international studies: the Alzheimer's Disease Neuroimaging Initiative (ADNI),^18^ the Australian Imaging, Biomarkers, and Lifestyle Flagship study of Ageing (AIBL),^19^ and the Open Access Series of Imaging Studies (OASIS).^20^ Each individual cohort included information on participant demographics, apolipoprotein E (*APOE)* ε4 carriage, and Clinical Dementia Rating (CDR) global score. Inclusion and exclusion criteria, along with a harmonization protocol for the cognitive and Aβ‐PET data are reported in detail elsewhere.^21^ Briefly, the criteria for the ADOPIC study required individuals to have complete neuropsychological test data for at least three assessments and excluded any individuals diagnosed with non‐AD syndromes such as Parkinson's disease, frontotemporal dementia, and vascular disease. The final overall ADOPIC sample included a total of 3658 participants across different clinical groups (CU, MCI, AD).

In this study, a subset of 1964 participants with full ADOPIC study cognitive data was created to conduct PCA and baseline analyses. This included participants who were CU (CDR global score of 0) at baseline, a subset of whom subsequently progressed to MCI (CDR global of 0.5). The harmonized nature of the ADOPIC dataset provided a comprehensive range of cognitive measures, extensive follow‐up, and a large sample size across three different cohorts. Finally, the inclusion criteria for the longitudinal subset required ≥3 assessments, resulting in a subset of 1110 participants (5297 observations), including 336 from ADNI, 575 from AIBL, and 199 from OASIS (see Figure S1 for more details on included participants).

### 2.2 Aβ‐PET quantification and *APOE* ε4 genotyping

The three cohorts of ADOPIC have used different Aβ‐PET tracers over the years, including ^18^F‐florbetaben, ^18^F‐florbetapir, ^18^F‐flutemetamol, ^18^F‐NAV4694, and ^11^C Pittsburgh compound B. Therefore, Aβ‐PET images were first smoothed, then spatially normalized using the standard statistical parametric mapping Centiloid (CL) pipeline. Then the images were normalized using capAIBL,^22^ a PET quantification pipeline, and the Aβ load in the brain was quantified using a Near Matrix Factorization Composite Universal values (CL values).^23^ Subsequently, baseline Aβ‐PET CL values were used in the analyses outlined below. Detailed descriptions regarding the CL transformation have been reported elsewhere.^22^ In line with previous works from the ADOPIC project, Aβ positivity (Aβ+) was defined as Aβ burden ≥ 20 CL.^24^ Genetic information included the *APOE* ε4 carriage status that included two classes: 0 – non‐carriers (ε3/ε3, ε2/ε2, ε3/ε2), and 1 – carriers (ε4/ ε2, ε4/ ε3, ε4/ ε4).

### 2.3 Cognitive measures

The ADOPIC cognitive database contains the tests listed in Table 1, indicating which measures were not used in all three studies (ADNI, AIBL, and OASIS) and therefore were subject to imputation. Complete information on the selection and harmonization of cognitive data was reported previously.^21^ Briefly, MissForest, a random forest‐based imputation approach, was used to harmonize cognitive test scores across the three cohorts. As a result, complete test scores (imputed) with no missing values were available for all participants across the tests listed in Table 1.^25^ To construct cognitive composites, a systematic approach was employed by selecting the most appropriate measures from this harmonized battery. This approach was guided by two main criteria: (1) avoiding redundancy in cognitive tests, that is, selecting either Rey Auditory Verbal Learning Test (RVLT) or California Verbal Learning Test, Second Edition (CVLT‐II) to represent verbal learning, and (2) prioritizing measures relevant to preclinical AD cognitive changes. For example, recognition measures from list learning were excluded from the analysis as they have been found to show ceiling effects and low reliability in CU individuals.^13^

**TABLE 1:** ADOPIC cognitive measures and their availability in the three cohorts.

Measure	ADNI	AIBL	OASIS
Mini‐Mental State Examination	**X**	**X**	**X**
Digit Symbol Coding	**X**	**X**	**X**
Logical Memory II (delayed recall)	**X**	**X**	**X**
Category Fluency Animals (total score)	**X**	**X**	**X**
Boston Naming Test (no cue recall)	**X**	**X**	**X**
Rey Auditory Verbal Learning Test (total, immediate, and delayed recall)	**X**		
California Verbal Learning Test, second edition (CVLT‐II) (total, immediate, and delayed recall)		**X**	
Buschke Selective Reminding Test (free recall)			**X**
Trail Making Test Part B	**X**		**X**
Digit Span Forward	**X**	**X**	**X**
Digit Span Backward	**X**	**X**	**X**
Verbal fluency switching (fruit and furniture)		**X**	

*Note*: X denotes whether the original test score is available in each cohort.

Abbreviations: ADNI, Alzheimer's Disease Neuroimaging Initiative; ADOPIC, Alzheimer's Dementia Onset and Progression in International Cohorts; AIBL, Australian Imaging, Biomarkers, Lifestyle, Flagship Study of Ageing; OASIS, Open Access Series of Imaging Studies.

### 2.4 Statistical analysis

All statistical analysis was performed using R version 4.2.3.^26^ Detailed descriptions of the packages used for the analysis can be found in the supplementary material.

To assess the effect of Aβ pathology at baseline (first visit), group differences between CU Aβ− and Aβ+ groups were assessed using the Wilcoxon rank‐sum test for continuous variables and Pearson's chi‐squared (χ2) tests for categorical variables. The magnitude of difference between the two groups for continuous variables was characterized using Cohen's *d*. This analysis included key demographic variables (age, sex, and education), *APOE* ε4 status, clinical progression status at 8.35 years (CU or MCI), time to progression status, and cognition indexed using cognitive composites detailed below, ADCS‐PACC, and MMSE.

#### 2.4.1 Composite development using PCA

Baseline (cross‐sectional) data of 1964 CU individuals were used in the PCA to develop cognitive composites in this study. Prior to the PCA, baseline cognitive test scores were transformed into z‐scores using the means and standard deviation (SD) of the baseline visit of the CU Aβ− group. The z‐scores were then entered into the PCA model to derive a lower‐dimensional orthogonal representation of the cognitive data.

The Kaiser‐Meyer‐Olkin (KMO) index was used to assess the sampling adequacy of the dataset. The PCA using the *psych* ^27^ package was carried out in three steps. First, varimax rotation was carried out on the principal components (PCs) to ensure the resulting PCs remained uncorrelated with each other.^28^ Second, parallel analysis and the Kaiser criterion (eigenvalues > 1) were used to guide the extraction of the number of PCs. Third, a loading threshold of ≥0.8^29^ was applied to select individual tests that have substantially contributed to each PC. This conservative threshold was chosen to ensure maximal domain purity and to reduce multicollinearity. Subsequently, the z‐scores of the tests significantly loading (item loading ≥ 0.8) onto each component were averaged to create a composite score, with each test weighted by its component loading^17^ and divided by the total number of tests loading onto the respective component (Equation 1). The coherence of test groupings within each component was confirmed by the ADOPIC Cognitive Stream team (including H.R.S, P.M., J.H., Y.Y.L., and R.S.), with reference to previous instances of PCA‐derived cognitive domain classification.^16^, ^17^, ^28^

Weighted composite score:

(1) $$\;\mathrm{Composite}\;\mathrm{Scor}e_{j}=\frac{\sum_{i=1}^{n}w_{i}x_{ij}}{n}.$$

where *n* = number of measures, *w_i_* = corresponding weights, *x_i_* = individual test score (z), and *j* = *j*th participant.

These steps resulted in domain‐specific composite scores representing distinct cognitive domains. Finally, tests of significant loading across all three PCs were combined into a general cognitive composite, using the same weighting approach (weighted by component loadings and divided by the total number of included tests, Equation 1). The resulting domain‐specific and general cognitive composites were compared against the MMSE and an approximation of the previously established ADCS‐PACC, a summed z‐score, using z‐scores from the following cognitive measures: Logical Memory II delayed recall, Buschke Selective Reminding Test free recall, Digit Symbol Coding, and the MMSE total score. The ADOPIC cognitive composites were then validated in a four‐step manner, noted in Sections 2.4.2 to 2.4.5.

#### 2.4.2 Psychometric examination of cognitive composites

The following analysis was conducted on the baseline visit data of 1964 CU older adults. First, generalized linear models (GLMs) with Type III sum of squares (SS), controlling for age, sex, education, and *APOE* ε4 status, were used to assess the discriminative validity of the cognitive composites between CU Aβ+ and CU Aβ− groups. Type III SS was used to ensure each predictor was evaluated after accounting for all others given known associations between Aβ, age, and *APOE* ε4 genotype. The magnitude of group differences was quantified using Cohen's *d*. Second, the clinical sensitivity of the composites was evaluated by examining group differences between CU Aβ+ and MCI Aβ+ groups, which were matched 1:1 using nearest‐neighbor matching (using *MatchIt* package) on age, sex, and education. Group differences were then assessed using the GLM with Type III SS with the same covariates. Internal consistency was evaluated using McDonald's omega total (*ω_t_*) for items within each domain‐specific composites and McDonald's omega hierarchical (*ω_h_*) and total for items within general composites. Distributional properties were assessed using skewness and kurtosis calculated at baseline for each composite. Floor and ceiling effects were calculated by examining the percentage of participants scoring at or near each cognitive composite's minimum and maximum values.

#### 2.4.3 Longitudinal sensitivity analysis

A longitudinal subset of 1110 participants who were CU at baseline were included for the longitudinal sensitivity analysis. The inclusion criteria are outlined in Section 2.1. and Figure S1. Linear mixed models (LMMs) using the *lme4* ^30^ and *lmerTest* ^31^ packages with a restricted maximum likelihood estimation were used to assess the sensitivity of the cognitive composites to baseline Aβ‐related cognitive decline. First, a base model with demographic features (baseline age, sex and education) and time (years since baseline visit) was established. Baseline Aβ status, *APOE* ε4 carriage, *APOE* ε4 carriage × time and baseline Aβ status × time were added incrementally. This approach allowed us to examine the incremental contribution of baseline Aβ status, *APOE* ε4, and aging (time), over and above demographic predictors, in estimating rates of cognitive change. An interaction term for baseline age by time was also included to examine whether rates of cognitive change varied as a function of baseline age, allowing the model to capture age‐dependent differences in longitudinal trajectories. All the models were adjusted for baseline age, sex, and education. Random slopes were included for time and random intercepts for participant ID with an unstructured covariance structure. Model assumptions were verified through visual inspection of the posterior predictive check, normality of the residuals, homogeneity of variance, and multicollinearity was assessed using the variance inflation factor (VIF) value. The incremental LMMs were compared using analysis of deviance and log‐likelihood ratio tests, and the final model's performance was indexed using the Akaike information criterion (AIC), Bayesian information criterion (BIC), and *R* ^2^ estimates.

Based on the recently published MAYO‐PACC^13^ work, Jackknife resampling was performed to assess sensitivity to Aβ‐related cognitive decline. The Jackknife resampling involved systematically removing one participant at a time from the total sample of 1110 participants, refitting the LMMs, and calculating the magnitude of difference (with standard error and confidence intervals) in the Aβ‐related slopes between each new cognitive composite, ADCS‐PACC, and the MMSE. This process was repeated for each participant to estimate the magnitude and confidence intervals of the differences (Δ). Statistical significance was determined by whether the 95% confidence intervals (CIs) included zero.

#### 2.4.4 Predicting time to progression to MCI

Cox proportional hazards regression using the *survival* package^32^ was used to assess the utility of the ADOPIC cognitive composite scores (developed in Section 2.4.1) at baseline in predicting time to MCI progression over 8.35 years (see Section 3.1 for justification for this follow‐up period). In this analysis, the baseline data of 1964 participants along with their status and time to progression/censoring were used in the modeling. Cox regression, a form of survival analysis, predicts the log‐relative hazard of an event (in this case, progression to MCI). Survival time was defined as the time between the first visit and the visit at which a participant was classified as MCI (CDR global score = 0.5). Participants who never met the criteria for MCI were censored at their last known visit. Because the event was defined specifically as progression to MCI, participants who transitioned directly from CU to dementia due to AD without an intervening MCI classification (*n* = 6) were treated as censored at their final pre‐AD assessment rather than as progressors. To confirm that this definition did not influence the findings, a sensitivity analysis was conducted in which the status was defined as first progression to MCI or dementia, reclassifying the six participants as progression events. Hazard ratios for all cognitive composites were within 0.02 of those reported in the primary analysis of this study, indicating that the progression definition did not affect the conclusions.

In the primary analysis, first, a base model was established, including baseline age (centered), sex, and education (centered). Baseline Aβ status, *APOE* ε4 allele carriage status, and cognitive composites were incrementally added as predictors. The AIC values from the forward stepwise selection approach were used to select the final model. Concordance index (C‐index) was used to assess discriminative ability. The final model formula was applied in six models, learning and memory, executive function, attention and processing speed, general cognition, ADCS‐PACC, and MMSE.

#### 2.4.5 Classification performance: Sensitivity and specificity analysis

To complement the Cox regression (Section 2.4.4), which quantified progression risk through hazard ratios, we evaluated the classification performance of the cognitive composites, adjusted for the same covariates (Section 2.4.4), using extreme gradient boosting (XGBoost).^33^, ^34^ Although XGBoost is typically applied in high‐dimensional settings, it was selected after it outperformed in a systematic comparison against logistic regression, random forest, and support vector machine classifiers (Figure S2). Furthermore, XGBoost's ability to capture potential non‐linear interactions between the predictors was considered an additional advantage over standard linear approaches. The following hyperparameters were identified through grid search cross‐validation: learning rate = 0.00186, tree depth = 5, minimum loss reduction = 9.42, minimal node size = 26, proportion of training observations sampled per tree = 0.306, number of features (predictors) = 6, number of iterations = 1237. This approach provides complementary validation to the predictive validity assessed in Section 2.4.4 by assessing sensitivity, specificity, and overall accuracy in distinguishing MCI progressors from CU stable. Due to the class imbalance (a disproportionate progressors to MCI vs CU stable individuals at 8.35 years), the minority class (MCI) in the training set was up‐sampled to address the class imbalance, in line with previous work in this area.^35^ The final dataset was divided into training (80%) and testing (20%) sets. Bootstrapped resamples (1000) were used to create the 95% CIs. Model performance on the test set was assessed using receiver operating characteristic area under the curve (ROC‐AUC), sensitivity, specificity, and positive and negative predictive values.

## 3 RESULTS

### 3.1 Preliminary analysis

Data obtained from the ADOPIC consortium for this study was characterized by extensive follow‐up data over 14 years. However, the analysis for this study was restricted to 8.35 years of available data for the following reasons: (1) Data for the OASIS cohort within ADOPIC concluded at 8.35 years, while ADNI and AIBL extended up to 12 and 14 years. Preliminary findings from a Kaplan‐Meier plot (Figure S3) revealed an accelerated decline in survival rates beyond 8.35 years, likely due to the selective continuation of only two cohorts (ADNI and AIBL). Therefore, restricting the follow‐up to 8.35 years ensured balanced representation across all three cohorts while maintaining sufficient duration for meaningful longitudinal analyses. (2) The 8.35‐year duration allowed for the modeling of cognitive changes over a long period using the cognitive composite scores.

However, to test the effect of various follow‐up restrictions on our longitudinal sensitivity analysis, different subsets of the ADOPIC data were modeled using LMMs. The different subsets of the data included 8.35 years from baseline, 5 years from baseline, 5 years before the last visit or visit at which a participant progressed to MCI, and the full dataset. The results showed that the estimates of various fixed effects (age, sex, education, *APOE* ε4, and time) and the interaction effect (*APOE* ε4 × time) from the LMMs were similar across the subsets. A detailed description of the findings from this preliminary analysis is presented in Figures S4–S7. In the longitudinal analysis, only participants with ≥3 observations and complete data (demographic, cognitive, genetic, and pathological) were included.

### 3.2 Baseline sample characteristics

The baseline visit data consisted of 1964 CU individuals. Participants were classified as Aβ− (*N* = 1345) or Aβ+ (*N* = 619) based on their baseline Aβ‐PET CL scores (Aβ+ ≥ 20 CL). Baseline sample characteristics are presented in Table 2. Group comparisons revealed that the CU Aβ+ group was older (mean difference = 2.53 years, p < 0.001) and had a higher number of *APOE* ε4 allele carriers than the CU Aβ− individuals. Sex and education levels were comparable between groups (both *p* > 0.05). Compared with the CU Aβ− group, the CU Aβ+ group showed a higher rate of progression to MCI (27% vs 12%) with a shorter time to progression (mean 3.01 vs 3.73 years). While group differences between CU Aβ− and Aβ+ were observed in the learning and memory, executive function, general cognition, MMSE, and ADCS‐PACC scores (all *p* < 0.001), the magnitude of these differences was small.

**TABLE 2:** Baseline sample characteristics of cognitively normal individuals by Aβ status.

Measure	Overall (*N* = 1964)	CU Aβ− (*N* = 1345)	CU Aβ+ (*N* = 619)	*p*	Cohen's *d* (95% CI)
Age	72.80 (6.40)	72.00 (6.32)	74.53 (6.24)	**<0.001**	−0.40 (−0.50 to ‐0.31)
Sex (F)	(1100) (56%)	(766) (57%)	(334) (54%)	0.2	—
Education (years)	14.75 (3.22)	14.75 (3.23)	14.75 (3.21)	>0.9	0.00 (−0.10 to 0.10)
*APOE* ε4 (carrier)	(603) (31%)	(296) (22%)	(307) (50%)	**<0.001**	—
Progressed to MCI	(334) (17%)	(168) (12%)	(166) (27%)	**<0.001**	—
Time to status (years)	3.50 (3.07)	3.73 (3.17)	3.01 (2.79)	**<0.001**	0.24 (0.14 to 0.33)
MMSE (max score = 30)	28.72 (1.29)	28.78 (1.24)	28.58 (1.37)	**0.002**	0.16 (0.07 to 0.26)
ADCS‐PACC (z)	−0.07 (0.72)	0.00 (0.69)	−0.21 (0.76)	**<0.001**	0.29 (0.19 to 0.38)
Learning and memory (z)	−0.07 (0.91)	−0.01 (0.89)	−0.19 (0.93)	**<0.001**	0.20 (0.11 to 0.30)
Executive function (z)	−0.09 (0.95)	−0.01 (0.90)	−0.27 (1.04)	**<0.001**	0.27 (0.18 to 0.37)
Attention and processing speed (z)	−0.01 (0.77)	−0.01 (0.78)	−0.02 (0.75)	0.7	0.02 (−0.08 to 0.11)
General cognition (z)	−0.06 (0.61)	−0.01 (0.59)	−0.16 (0.66)	**<0.001**	0.25 (0.16 to 0.35)

*Note*: For continuous variables, mean and SD were reported. Group differences were carried out using the Wilcoxon rank‐sum test, and the effect size was calculated using Cohen's *d*. For categorical variables, percentages were reported, and group differences were calculated using chi‐squared statistic. *p* values bolded if significant at *p* < 0.05. All cognitive composites (learning and memory, executive function, attention and processing speed, general cognition, and ADCS‐PACC) and MMSE are z‐scores with a mean of 0 and SD of 1. A higher score on cognitive composites and MMSE reflects better cognition.

Abbreviations: 0 – non‐progressor (CU), 1 – progressor (MCI); ADCS‐PACC, Alzheimer's Disease Cooperative Study, Preclinical Alzheimer's Cognitive Composite; APOE e4, apolipoprotein E ε4 allele; CU Aβ−, cognitively normal amyloid beta negative; CU Aβ+, cognitively normal amyloid beta positive; F, female; M, male; MCI, mild cognitive impairment; MMSE, Mini‐Mental State Examination; progression status (over 8.35 years).

### 3.3 Cognitive composite development using PCA

The varimax‐rotated loadings of the individual PCs are displayed in Figure 1. The KMO statistic of 0.73 suggested adequate sampling adequacy for the PCA.^36^ Results from parallel analysis and eigenvalue convergence suggested a three‐component solution, which together accounted for 64.3% of the total variance in cognitive performance at baseline. Individual tests of significant loading (>0.8) on PC1 reflected cognitive processes underlying learning and memory (Table 3). Similarly, tests loading on PC2 and PC3 reflected executive function and attention and processing speed, respectively (Table 3). The component loadings from the tests loading above 0.8 for each PC were extracted and used as item weights to create three domain‐specific composite scores, which were then combined to form a general cognitive composite score. Furthermore, sensitivity analysis confirmed that weighted and unweighted composites were perfectly correlated (r = 1.00) for all composites, reflecting a high degree of domain purity within each PC and indicating that differential weighting had no practical impact on composite scores. Therefore, four cognitive composites were calculated for each participant in the sample: learning and memory, executive function, attention and processing speed, and general cognition.

**FIGURE 1:**
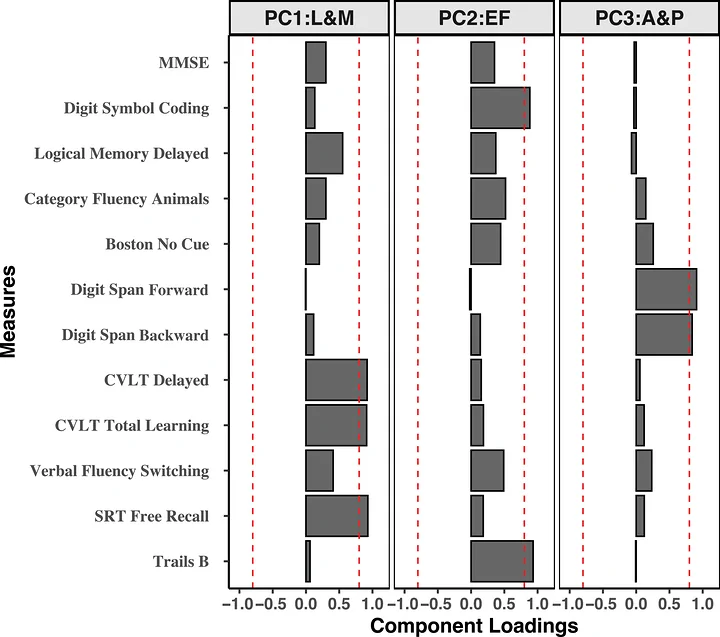
Principal component analysis loading plot. Component loadings plot depicting individual item loadings on each of the principal components. Component loading threshold (indicated by red dotted line) was set at 0.8 to ensure maximum domain purity. Individual principal components named after the cognitive processes reflected by the significant loading tests. L&M, learning and memory; EF, executive function; A&P, attention and processing speed; MMSE, Mini‐Mental Status Examination; DSCD, digit symbol coding; LMII_Del, logical memory II delayed recall; TMT_B, Trail Making Test Part B; CFLU_Anim, category fluency (animals); BOST_NoCue, Boston Naming Test (no cue recall); DSFW, digit span forward; DSBW, digit span backward; CVLT_Del, California Verbal Learning Test (delayed recall); VFLU_Swi, verbal fluency (switching); SRTFREE, Buschke Selective Reminding Test (free recall).

**TABLE 3:** ADOPIC domain‐specific and general cognitive composite scores components.

Composite score	Tests and associated weights
Learning and memory	(0.920 × CVLT‐II Delayed + 0.912 × CVLT‐II Total + 0.922 × SRT Free Recall) ÷ 3
Executive function	(0.898 × Digit Symbol Coding + 0.941 × TMT B) ÷ 2
Attention and processing speed	(0.906 × Digit Span Forward + 0.846 × Digit Span Backward) ÷ 2
General cognition	(0.920 × CVLT‐II Delayed + 0.912 × CVLT‐II Total + 0.922 × SRT Free Recall + 0.898 × Digit Symbol Coding + 0.941 × TMT B + 0.906 × Digit Span Forward + 0.846 × Digit Span Backward) ÷ 7

Abbreviations: CVLT, California Verbal Learning Test; SRT, Buschke Selective Reminding Test free recall; TMT B, Trail Making Test B.

### 3.4 Validation of cognitive composites

Criterion validity analysis using GLM, controlling for age, sex, and education, revealed differential patterns of group differences across various comparisons (Figure 2). First, at baseline in CU individuals, small non‐significant differences between Aβ− and Aβ+ groups were observed for all ADOPIC composites (*d* = −0.03 to 0.18, all *p* > 0.12). Only the ADCS‐PACC (*F *= 5.94, *p *= 0.015, *d* = 0.22, 95% CI = 0.12 to 0.33) and MMSE (*F* = 8.68, *p* = 0.003, *d *= 0.19, 95% CI = 0.08 to 0.30) showed significant group differences. Effect sizes for all composites are presented in Figure 2A. Second, discriminative validity analysis of CU Aβ+ and MCI Aβ+ (those who progressed to MCI at a later date) individuals (matched on age, sex, and education) revealed group differences across all ADOPIC composites and ADCS‐PACC, while MMSE showed no meaningful discrimination. General cognition demonstrated the strongest discrimination (*F* = 25.41, *p* < 0.001, *d* = 0.55, 95% CI = 0.32 to 0.77), followed by learning and memory (*F* = 17.20, *p* < 0.001, *d* = 0.47, 95% CI = 0.24 to 0.70), ADCS‐PACC (*F* = 14.03, *p* < 0.001, *d* = 0.42, 95% CI = 0.19 to 0.64), executive function (*F* = 11.01, *p* < 0.001, *d* = 0.36, 95% CI = 0.13 to 0.58), and attention and processing speed (*F* = 8.04, p < 0.01, *d* = 0.30, 95% CI = 0.07 to 0.52). Effect sizes for all composites are presented in Figure 2B.

**FIGURE 2:**
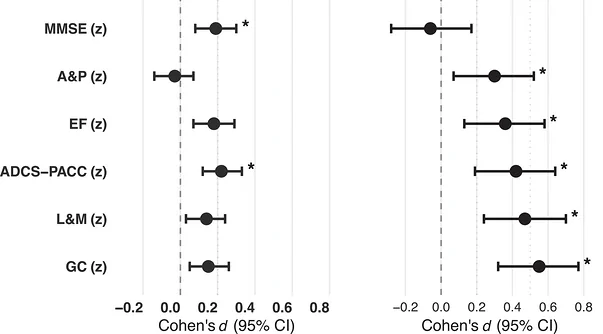
Discriminative validity of ADOPIC and comparator composites. Effect sizes (Cohen's *d*) with 95% confidence intervals from the GLM models controlling for age, sex, education, and *APOE* ε4 status. (A) Baseline discrimination between cognitively unimpaired amyloid‐positive (CU Aβ+) and amyloid‐negative (CU Aβ−) individuals. (B) Clinical sensitivity comparing matched CU Aβ+ and MCI Aβ+ individuals (1:1 nearest‐neighbor matching on age, sex, and education). Vertical dashed lines indicate null effect (*d* = 0); dotted lines indicate small (*d* = 0.2) and medium (*d* = 0.5) effect size benchmarks. Asterisks (*) indicate *p* < 0.05. MMSE (z), Mini‐Mental State Examination z‐score; A&P (z), attention and processing speed composite z‐score; EF (z), executive function composite z‐score; ADCS‐PACC (z), Alzheimer's Disease Cooperative Study – Preclinical Alzheimer's Cognitive Composite z‐score; L&M (z), learning and memory composite z‐score; GC (z), general cognition composite z‐score.

The internal consistency analysis using McDonald's omega showed that general cognition demonstrated excellent overall reliability (*ω_t_* = 0.94), with omega hierarchical analysis revealing that 62% of the variance was attributable to a general cognitive factor (*ω_h_* = 0.62) and 31% to domain‐specific factors (*ω*_group = 0.31), supporting a hierarchical structure encompassing both general and domain‐specific cognitive abilities. All four cognitive composites were approximately normally distributed (skewness range = −0.81 to 0.61; ordinary kurtosis range = 2.50 to 3.57). Analysis of distributions showed the following skewness and kurtosis values: learning and memory (skewness: −0.384, kurtosis: 2.50), executive function (skewness: −0.807, kurtosis: 3.57), attention and processing speed (skewness: 0.611, kurtosis: 3.16), ADCS‐PACC (skewness: −0.42, kurtosis: 3.08), and general cognition (skewness: −0.328, kurtosis: 2.96). While other cognitive composites displayed negative skewness, attention and processing speed showed positive skewness (0.611) with a broader range toward higher values (ceiling of 2.89), suggesting a different distributional pattern for attention abilities compared to other cognitive domains. However, all measures remained within acceptable normality parameters. Examining floor and ceiling effects revealed that only 0.05% of participants scored at the minimum (learning and memory: −3.13, executive function: −3.9, attention and processing speed: −1.88, general cognition: −2.53) or maximum (learning and memory: 1.81, executive function: 2.28, attention and processing speed: 2.89, general cognition: 1.84) values for each composite, indicating good distributional characteristics for the ADOPIC cognitive composites.

### 3.5 Sensitivity to longitudinal Aβ‐related cognitive decline

Model assumptions for all the LMMs were assessed through visual inspection of diagnostic plots (Figures S8–S11). All models showed good posterior predictive fit and low multicollinearity (all VIF < 2), with minor deviations from normality and homoscedasticity, to which LMMs have been reported to be robust.^37^ Model selection using analysis of deviance and log‐likelihood ratio tests indicated that the final model, including baseline age × time, *APOE* ε4 carriage × time, and Aβ status × time interactions, provided the best fit across all cognitive measures (Tables S1–S4). Intraclass correlation coefficients (ICCs) indicated that between 51% and 79% of variance in cognitive composite scores was attributable to stable between‐person differences (ICC range = 0.40 to 0.79), with MMSE showing the lowest ICC (0.40) and general cognition the highest (0.79).

Estimates of various fixed and random effects from the LMMs for the four cognitive composites, ADCS‐PACC, and MMSE are presented in Table 4. Older age at baseline was associated with poorer cognition across the six cognitive composites, female sex was associated with better cognition across all models except attention and processing speed, and more years of education were associated with better cognition across five models with the exception of the attention and processing speed composite. The fixed effect of time varied across composites (Table 4). Learning and memory showed a positive but non‐significant time coefficient, and executive function, attention and processing speed, and general cognition showed significant negative time effects in the CU Aβ− reference group. ADCS‐PACC and MMSE showed no significant time effects (*p* > 0.05). All six cognitive composites showed sensitivity (*p* < 0.05) to baseline Aβ‐related cognitive decline, confirming known group differences in the cognitive trajectories of CU Aβ− and + groups (Figure 3). While the interaction effects between time and baseline age and between time and *APOE* ε4 carriage were significant (*p* < 0.05) in learning and memory and general cognition, it was not significant for other models. The model fit was comparable in the three domain‐specific cognitive composites. The models with the best fit were those of general cognition, that is, general cognition and ADCS‐PACC. The model with the poorest fit, that is, the highest AIC value, was the MMSE model.

**TABLE 4:** Estimates of fixed and random effects from linear mixed models.

	Learning and memory	Executive function	Attention and processing speed	General cognition	ADCS‐PACC	MMSE
Fixed and interaction effects	β (95% CI)	β (95% CI)	β (95% CI)	β (95% CI)	β (95% CI)	β (95% CI)
Baseline age (reference: 72.6 years)	**−0.03** ^***^ (−0.04 to −0.02)	**−0.04** ^***^ (−0.05 to −0.03)	**−0.02** ^***^ (−0.03 to −0.01)	**−0.03** ^***^ (−0.03 to −0.02)	**−0.02** ^***^ (−0.03 to −0.02)	**−0.01** ^**^ (−0.02 to −0.01)
Time (years)	0.01 (−0.00 to 0.02)	**−0.04** ^***^ (−0.05 to −0.03)	**−0.01** ^*^ (−0.01 to −0.00)	**−0.01** ^*^ (−0.01 to −0.00)	−0.00 (−0.01 to 0.01)	−0.01 (−0.03 to 0.01)
Sex (Male)	**−0.51** ^***^ (−0.61 to −0.42)	**−0.16** ^***^ (−0.25 to −0.07)	**0.08** ^*^ (0.00 to 0.17)	**−0.24** ^***^ (−0.31 to −0.18)	**−0.33** ^***^ (−0.40 to −0.26)	**−0.18** ^***^ (−0.27 to −0.09)
Education (reference: 14.6 years)	**0.05** ^***^ (0.03 to 0.06)	**0.10** ^***^ (0.09 to 0.11)	**−0.02** ^*^ (−0.03 to −0.00)	**0.04** ^***^ (0.03 to 0.05)	**0.08** ^***^ (0.06 to 0.09)	**0.05** ^***^ (0.04 to 0.07)
*APOE* ε4 (carriers)	**−0.15** ^**^ (−0.26 to −0.04)	−0.05 (−0.16 to 0.05)	−0.06 (−0.16 to 0.04)	**−0.09** ^*^ (−0.17 to −0.02)	−0.04 (−0.12 to 0.04)	0.01 (−0.10 to 0.13)
Baseline Aβ status	−0.06 (−0.17 to 0.05)	−0.08 (−0.19 to 0.03)	0.01 (−0.09 to 0.11)	−0.04 (−0.11 to 0.03)	−0.07 (−0.15 to 0.01)	−0.02 (−0.13 to 0.10)
Baseline age‐centered × time	−**0.00** ^***^ (−0.01 to −0.00)	**−0.01** ^***^ (−0.01 to −0.00)	−0.00 (−0.00 to 0.00)	**−0.00** ^***^ (−0.00 to −0.00)	**−0.01** ^***^ (−0.01 to −0.00)	**−0.01** ^***^ (−0.01 to −0.00)
*APOE* ε4 carriage × time	**−0.03** ^**^ (−0.05 to −0.01)	**−0.02** ^*^ (−0.04 to −0.00)	−0.01 (−0.02 to 0.00)	**−0.02** ^**^ (−0.03 to −0.01)	**−0.03** ^***^ (−0.05 to −0.02)	**−0.03** ^*^ (−0.06 to −0.00)
Baseline Aβ status × time	**−0.07** ^***^ (−0.09 to −0.05)	**−0.05** ^***^ (−0.07 to −0.03)	**−0.02** ^***^ (−0.04 to −0.01)	**−0.05** ^***^ (−0.07 to −0.04)	**−0.07** ^***^ (−0.09 to −0.05)	**−0.08** ^***^ (−0.11 to −0.05)

Random effects	Learning and memory	Executive function	Attention and processing speed	General cognition	ADCS‐PACC	MMSE
SD of intercept	0.70	0.69	0.66	0.47	0.52	0.55
SD of slope	0.09	0.06	0.02	0.06	0.07	0.10
Correlation of intercept and slope	0.14	0.10	−0.04	0.09	0.12	0.01
ICC	0.75	0.71	0.76	0.79	0.77	0.40
Marginal *R* ^2^/Conditional *R* ^2^	0.17/0.79	0.26/0.78	0.04/0.76	0.25/0.84	0.27/0.83	0.09/0.45

*Note*: All units are z‐scores. Age was centered at 72.6 years; education was centered at 14.6 years. Female participants, Aβ – and *APOE* ε4 non‐carriers were the reference levels.

Abbreviations: ADCS‐PACC, Alzheimer's Disease Cooperative Study – Preclinical Alzheimer Cognitive Composite; MMSE, Mini‐Mental State Examination; *APOE* ε4, apolipoprotein E ε4 allele; Aβ, amyloid beta; ICC, intraclass correlation coefficient.

* *p* < 0.05.

** *p* < 0.01.

*** *p* < 0.001.

**FIGURE 3:**
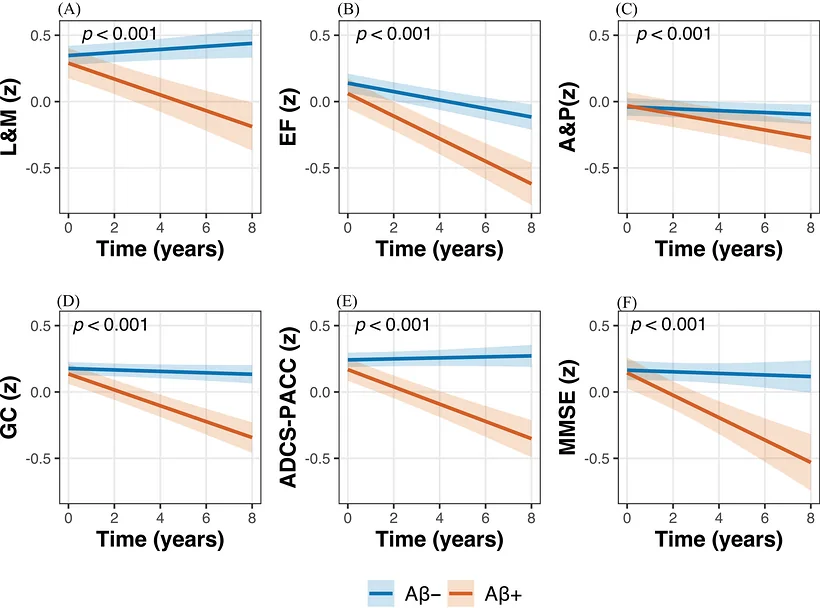
Cognitive composite trajectories stratified by baseline Aβ status. Predicted trajectories are derived from linear mixed‐effects models with random intercepts and slopes for time. Predictions are shown for a reference participant (female, *APOE* ε4 non‐carrier, education [centered], baseline age [centered]). Shaded regions represent 95% confidence intervals. *p* values reflect the baseline Aβ status by time interaction term. All composites are z‐scores with a mean of 0 and a stand deviation of 1. (A) L&M (z), learning and memory composite z‐score. (B) EF (z), executive function composite z‐score. (C) A&P (z), attention and processing speed composite z‐score. (D) GC (z), general cognition composite z‐score. (E) ADCS‐PACC, Alzheimer's Disease Cooperative Study‐Preclinical Alzheimer Cognitive Composite z‐score. (F) MMSE (z), Mini‐Mental State Examination z‐score. Aβ−, amyloid‐beta negative; Aβ+, amyloid‐beta positive.

Effect sizes for amyloid‐related slope differences are presented in Figure S12. Cohen's *d* values ranged from −0.062 (95% CI = −0.094 to −0.029) for attention and processing speed to −0.201 (95% CI = −0.249 to −0.153) for ADCS‐PACC. All cognitive composites showed statistically significant differences for Aβ‐related slope differences (all *p* < 0.001). Annual rates of decline between CU Aβ− and CU Aβ+ groups were as follows: learning and memory (0.009 vs −0.063), executive function (−0.036 vs −0.089), attention and processing speed (−0.007 vs −0.031), general cognition (−0.008 vs −0.062), ADCS‐PACC (0.000 vs −0.069), and MMSE (−0.013 vs −0.110).

Compared to the ADCS‐PACC, general cognition captured a slightly smaller Aβ‐related slope difference (Δ = 0.014, S.E = 0.005, 95% CI = 0.002 to 0.026). Learning and memory (Δ = −0.002, S.E = 0.008, 95% CI = −0.019 to 0.014) and executive function (Δ = 0.016, S.E = 0.009, 95% CI = −0.002 to 0.034) did not differ from ADCS‐PACC, indicating comparable performance in distinguishing the longitudinal trajectories of CU Aβ− and CU Aβ+ individuals. Finally, attention and processing speed was considerably less sensitive to Aβ‐related cognitive decline than the ADCS‐PACC, which showed the larger Aβ‐related slope difference (Δ = 0.046, S.E = 0.009, 95% CI = 0.026 to 0.064).

### 3.6 Predicting time to progression to MCI

Results from the Cox regression model building are presented in Table S5. Older age at baseline was associated with increased hazard of progressing to MCI across most models (Table 5), with the exception of the general cognition model, where the effect was non‐significant. A one unit increase in the baseline age was associated with a 2% to 5% increase in the hazard of progression to MCI, depending on the composite model. Unexpectedly, each additional year of education was associated with a 4% increase in the hazard of progression, but only in the ADCS‐PACC model. Although the direction of the effect of education seems counterintuitive, this may reflect a suppression effect, that is, education acts as a suppressor variable when ADCS‐PACC is in the model. The effect of education in the base model (not shown here) was negative, albeit insignificant, suggesting a 3% lower hazard of progression with each additional year of education. Baseline Aβ positivity was associated with a two‐fold increased hazard of progression to MCI.^38^ *APOE* ε4 allele carriage was not associated with progression to MCI across the three models. This could be due to the collinearity *(p* < 0.05) we observed between *APOE* ε4 carriage and baseline Aβ status, wherein the direct effect of *APOE* ε4 is attenuated when Aβ status is included in the model.

**TABLE 5:** Hazard ratios associated with measures for predicting progression to MCI.

Predictor	Learning and memory	Executive function	Attention and processing speed	General cognition	ADCS‐PACC	MMSE
	H.R. (95% CI)	H.R. (95% CI)	H.R. (95% CI)	H.R. (95% CI)	H.R. (95% CI)	H.R. (95% CI)
Baseline age (centered at 72.6)	**1.03** ^**^ (1.01 to 1.05)	**1.04** ^***^ (1.02 to 1.06)	**1.04** ^***^ (1.02 to 1.06)	1.02 (1.00 to 1.04)	**1.02** ^*^ (1.01 to 1.04)	**1.05** ^***^ (1.03 to 1.07)
Sex (male)	0.82 (0.65 to 1.04)	1.01 (0.80 to 1.28)	1.12 (0.89 to 1.42)	0.87 (0.69 to 1.10)	0.82 (0.64 to 1.03)	0.98 (0.78 to 1.25)
Education (centered at 14.8)	1.00 (0.96 to 1.03)	1.00 (0.97 to 1.04)	**0.96** ^*^ (0.93 to 0.99)	1.00 (0.97 to 1.04)	**1.04** ^*^ (1.00 to 1.08)	0.99 (0.96 to 1.02)
Baseline Aβ status	**2.05** ^***^ (1.60 to 2.62)	**2.15** ^***^ (1.68 to 2.75)	**2.20** ^***^ (1.72 to 2.81)	**1.98** ^***^ (1.55 to 2.54)	**2.01** ^***^ (1.57 to 2.57)	**2.15** ^***^ (1.68 to 2.75)
*APOE* ε4 (carriers)	1.20 (0.94 to 1.55)	1.19 (0.93 to 1.54)	1.24 (0.97 to 1.60)	1.19 (0.93 to 1.54)	1.18 (0.91 to 1.52)	1.21 (0.94 to 1.56)
Cognitive composite (z)	**0.42** ^***^ (0.36 to 0.50)	**0.77** ^***^ (0.67 to 0.87)	**0.65** ^***^ (0.55 to 0.77)	**0.34** ^***^ (0.28 to 0.42)	**0.40** ^***^ (0.34 to 0.48)	**0.75** ^***^ (0.67 to 0.83)
C‐index (SE)	0.72 (0.01)	0.65 (0.01)	0.67 (0.01)	0.71 (0.01)	0.71 (0.01)	0.67 (0.01)

*Note*: Hazard ratios (HR) with 95% confidence intervals reported for each predictor. HR > 1 indicates increased risk of progression, while HR < 1 indicates a protective effect. Sex (female), *APOE* ε4 (0) (non‐carriers), Aβ− were used as reference levels. Each model consisted of the composites combined with age, sex, education, Aβ, and *APOE* ε4 carriage status. Concordance index is reported along with the standard error (SE).

Abbreviations: ADCS‐PACC, Alzheimer's Disease Cooperative Study – Preclinical Alzheimer Cognitive Composite; APOE (4 [0], non‐carriers; APOE (4 [1], carriers; Aβ, amyloid beta, (−) negative, (+) positive; C‐index, concordance index; HR, hazard ratio; MMSE, Mini‐Mental State Examination.

* *p* < 0.05.

** *p* < 0.01.

*** *p* < 0.001.

Higher scores on baseline cognitive composite scores were significantly associated (all *p* < 0.001) with a reduced hazard of progression to MCI across composites (Table 5). The magnitude of the protective effect varied across composites. A one‐unit increase in the general cognition score showed the largest protective effect (66%), followed by ADCS‐PACC (60%), learning and memory (58%), attention and processing speed (35%), MMSE (25%), and executive function (23%). All six models showed acceptable to good discrimination, with C‐index ranging from 0.65 (executive function) to 0.72 (learning and memory).

### 3.7 Classification performance: sensitivity and specificity

Classification performance was evaluated using XGBoost across all six predictor combinations. The full results are reported and illustrated in Table S6. General cognition demonstrated the strongest overall performance (ROC‐AUC = 0.70, sensitivity = 0.63, specificity = 0.68), closely followed by the ADCS‐PACC (ROC‐AUC = 0.70, sensitivity = 0.57, specificity = 0.72). While general cognition showed higher sensitivity in classification, the ADCS‐PACC showed marginally higher specificity. Both general cognition and ADCS‐PACC showed comparable positive predictive values. Notably, both measures outperformed MMSE across all the metrics. Among domain‐specific composites, learning and memory achieved the highest ROC‐AUC (0.70), comparable to both general cognitive composites above. In comparison, executive function and attention and processing speed showed more modest discrimination with ROC‐AUC of 0.66. Notably, negative predictive values were consistently high across all composites, ranging from 0.88 to 0.90. As such, up‐sampling the minority class (MCI progressors) consistently improved sensitivity and specificity across all composites. Bootstrap validation (1000 resamples) confirmed the stability of these estimates, with standard errors of 0.001 to 0.002 and 95% CIs spanning less than 0.005 units for ROC‐AUC across all models, indicating reliable predictive performance.

## 4 DISCUSSION

### 4.1 Principal findings and contributions

In this work, we developed and validated data‐driven cognitive composites for CU older adults using PCA. The three PCs converged with domains covered by previous theory‐driven composites,^39^ providing empirical confirmation of established cognitive domains and evidence for structural validity for our composites. In contrast to our hypothesis, the four ADOPIC composites performed comparably to the ADCS‐PACC in terms of sensitivity to Aβ‐related cognitive decline and predicting progression to MCI. However, all four ADOPIC cognitive composites demonstrated good distributional properties and good to excellent internal consistency. Criterion validity analyses revealed differential sensitivity across disease stages. At baseline cross‐sectional differences between CU Aβ− and CU Aβ+ groups was non‐significant for all ADOPIC composites. This is in line with previously developed composites such as Mayo‐PACC and PACC‐Revised, which also did not show baseline group differences^13^ and comparisons of individual cognitive tests in our baseline sample (Table S7). In contrast, all ADOPIC composites showed robust differences CU‐stable‐Aβ+ and CU‐progressors‐Aβ+ (progressed to MCI at follow‐up).

This work makes three key contributions to previous findings. First, while previous data‐driven approaches demonstrated improvements in sensitivity to longitudinal Aβ‐related decline (magnitude of longitudinal cognitive decline associated with brain Aβ), our findings extend this to a larger multi‐cohort harmonized database.^13^, ^14^, ^40^ Second, we provide detailed psychometric reporting as possible within the scope of the data, addressing a well‐documented gap,^13^, ^14^, ^40^ including criterion validity, predictive validity, structural validity, internal consistency, and distributional properties. Third, by developing both domain‐specific and general composites, we provide a multidimensional assessment framework that may capture a range of heterogeneous cognitive manifestations of preclinical AD.

### 4.2 Advantages of the empirical approach to composite development

The empirical approach to composite development offers several advantages over a theory‐driven approach. First, the component structure and loadings from PCA can guide test selection by identifying which measures capture the most variance in baseline cognitive performance, thereby reducing redundancy and improving measurement efficiency.^3^, ^8^ Second, PCA provides explicit evidence for structural validity, a fundamental psychometric property that has frequently been overlooked in cognitive composite development.^14^, ^41^, ^42^ While a data‐driven approach was used to select tests and optimize their item weights, it must be noted that the test batteries used in ADOPIC studies were chosen based on theoretical findings, and the item weights derived from the PCA are specific to the sample of participants at baseline.

### 4.3 Sensitivity to preclinical AD pathology

Sensitivity to AD pathology is a critical benchmark for cognitive outcome measures used in observational studies and preclinical AD trials.^10^, ^40^ All four ADOPIC composites demonstrated sensitivity to Aβ‐related cognitive decline over 8 years, with general cognition and ADCS‐PACC showing the largest effect sizes of all. The differential sensitivity across cognitive domains provides insights into the heterogeneous cognitive phenotypes of preclinical AD.^43^ Learning and memory's longitudinal sensitivity (*d* = −0.15) confirmed episodic memory's established role as one of the early markers of AD‐related cognitive dysfunction.^1^, ^6^ Executive function demonstrated moderate sensitivity, consistent with the evidence that dysexecutive changes emerge in some individuals with preclinical AD progressing to MCI.^44^ Attention and processing speed showed the weakest longitudinal sensitivity (*d* = −0.06), potentially reflecting either true sparing or measurement limitations (two‐item test). Additionally, findings suggest that language and semantic memory show changes in preclinical AD.^45^, ^46^ However, measures of language and verbal fluency within the ADOPIC cognitive tests did not load onto any of the three PCs. This may reflect the sample used in the current study, whose variation was primarily captured across the three reported domains. Therefore, our PCA findings are representative of the current sample. As such, we acknowledge that assessment of language and verbal fluency in preclinical AD remains important. Finally, while no functional imaging analysis was included here, the differential domain sensitivity observed here is consistent with prior evidence that early Aβ accumulation preferentially affects default mode network regions supporting memory, executive function, and attentional control,^47^, ^48^, ^49^ generating hypotheses for future work

### 4.4 Comparisons with ADCS‐PACC

Comparisons between general cognition and ADCS‐PACC revealed both convergence and divergence. General cognition converged with the ADCS‐PACC in terms of comparable longitudinal sensitivity to Aβ‐related decline, supporting the continued use of established composites such as the ADCS‐PACC in preclinical AD trials, while also demonstrating that empirically derived alternatives offer complementary advantages in terms of domain‐specific measurement. Specifically, the ADOPIC composites offer empirically derived test selection, explicit evidence of structural validity, psychometric characterization across multiple domains, and performance across harmonized multi‐cohort data supporting generalizability. Findings diverged in baseline discrimination; neither ADOPIC composites nor ADCS‐PACC showed group differences after adjusting for covariates. MMSE alone showed baseline differences, likely reflecting a known ceiling effect, reducing sensitivity to subtle change. Likewise, the subset of CU individuals scoring below ceiling may be disproportionately likely to be Aβ+, potentially inflating cross‐sectional effect sizes. This is consistent with previous findings^6^, ^11^, ^13^ showing the MMSE's ceiling effects in a CU population, a slight change in the score could drive larger differences in effect sizes. Bransby et al.^11^ demonstrated that while the removal of MMSE from the ADCS‐PACC reduced the magnitude of the difference between Aβ− and Aβ+ groups, it did not improve the sensitivity to Aβ‐related cognitive decline. This pattern aligns with our findings and suggests MMSE's contribution to ADCS‐PACC may be primarily cross‐sectional. Conversely, general cognition showed a greater magnitude of discrimination of MCI progressors versus CU stable at baseline, suggesting it captures a broader range of cognitive dysfunction. Additionally, general cognition also showed higher internal consistency compared to that of the ADCS‐PACC, suggesting that empirical approaches to general composite development can produce a psychometrically robust measure.

### 4.5 Strengths and limitations

This study has several notable strengths, including a large harmonized multi‐cohort sample, standardized Aβ‐PET quantification, comprehensive validation, and an 8.35‐year follow‐up spanning the preclinical‐to‐prodromal continuum.

Nonetheless, there are several limitations that should be considered. First, composites were derived and validated on the same baseline sample without a hold‐out sample or cross‐validation, which may limit generalizability. Second, Aβ‐PET was only available at baseline for the current study, limiting examination of Aβ accumulation over time in relation to cognitive trajectories.^13^ Third, differences in assessment schedules across cohorts precluded both year‐by‐year sensitivity analyses and formal practice effect assessment, meaning we cannot determine when composites are most sensitive to Aβ‐related decline or identify optimal assessment windows. Fourth, the ADOPIC sample is predominantly Caucasian, highly educated, and English speaking, limiting the generalizability to diverse samples. Fifth, comparisons were restricted to ADCS‐PACC, which was developed specifically for the CU populations with evidence of AD pathology. While this comparison was informative in assessing the performance of our composites, additional comparisons such as Alzheimer's Disease Assessment Scale‐Cognitive Subscale were not feasible. Additionally, clinical status (CU or MCI) was defined using the CDR. Although this convention is standard and permits comparison with other studies, it is likely that some individuals classified as unimpaired by the CDR may exhibit objective cognitive impairment but have not yet noticed it in their lives. Likewise, a CDR of 0.5 for defining MCI may have excluded participants with subjective cognitive concerns but normal cognition on objective testing.^50^ Sixth, practical implementation considerations like administration time, proprietary measures, and clinician training requirements were not evaluated, which are critical for translation to clinical settings. Seventh, the two‐item composites may have affected their reliability, so future work should examine whether expanding with additional measures in the overall battery improves psychometric properties. Finally, validation was limited to Aβ‐PET and *APOE* ε4; future work should examine associations with tau‐PET and plasma biomarkers, given evidence that Aβ‐related cognitive dysfunction may be mediated by tau pathology and vascular changes.^48^

The findings of the current study may have potential clinical applications for screening assessments. Prevention trials in asymptomatic Aβ+ anticipating progression to MCI would benefit from measures like general cognition, with adequate range to detect meaningful worsening with reduced required sample size requirements. However, these suggestions must be interpreted with caution as they require developing normative data, determining clinically meaningful thresholds, feasibility in clinical practice, and power analysis, and work extending beyond the current study's scope. Nonetheless, there is work under way from various cohorts to assess the feasibility of using cognitive composite measures in clinical practice.^13^

## 5 CONCLUSION

In conclusion, we developed and validated four cognitive composites for assessing cognitive dysfunction in preclinical AD using a data‐driven approach applied to harmonized data. The composites demonstrated good psychometric properties, sensitivity to Aβ‐related cognitive decline, and the ability to predict progression to MCI. While external validation work remains, these cognitive composites are viable options for studies tracking cognitive decline in preclinical AD. Future work leveraging these composites will examine how domain‐specific cognitive performance relates to Aβ burden across the preclinical spectrum and whether early cognitive assessment can predict who is at risk for progression toward Aβ positivity. As prevention trials increasingly target earlier disease stages, the need for sensitive and reliable measures of subtle cognitive changes remains. The ADOPIC cognitive composites address this need while providing a foundation for deeper investigation into the nature and heterogeneity of preclinical AD cognitive decline.

## CONFLICT OF INTEREST STATEMENT

H.R.S. and R.N.M. report being directors of SMarT Minds WA, Australia. H.R.S. and R.N.M. have received or are receiving personal reimbursements or research support from pharmaceutical and nutraceutical companies including Alector, Alnylam Pharmaceuticals, CWEKPTYLTD, WA, Australia, and Biogen Pharmaceuticals. C.L.M. reports ad hoc consultancy speaking engagements and scientific advice with Actinogen, Acumen, Alterity, Biogen, Eisai, Eli Lilly, and Roche. C.C.R. has received research grants from National Health and Medical Research Council (NHMRC) of Australia, Medical Research Future Fund (MRFF), Enigma Australia, Biogen, Eisai, Roche, and AbbVie. He is on the scientific advisory board for Enigma Biomedical Group and consulted for Prothena, Roche, Eisai, Eisai Australia, Eli Lilly Australia, and Novo Nordisk Australia. M.W. serves on editorial boards for *Alzheimer's & Dementia* and the *Journal for Prevention of Alzheimer's Disease*. M.W. has provided consulting to Acadia Pharmaceuticals, Acumen Pharmaceuticals, Boxer Capital, BrightFocus Foundation, Cerecin, Clario/BioClinica, Dementia Society of Japan, Eisai, Guidepoint, Health and Wellness Partners, Indiana U., LCN Consulting, Merck Sharp & Dohme Corp., Duke University NC Registry for Brain Health, Owkin France, Novo Nordisk, ProMIS Neurosciences, Inc., Prova Education, Sai Med Partners, T3D Therapeutics, University of Southern California (USC), WebMD, MEDA Corp., Quantum Leap Health, REGEnLIFE, GLG Consulting, IXICO, Danaher, Medspoke, Splink, Inc., Mitre, and CervoMed. He also serves on the USC ACTC grant, which receives funding from Eisai. M.W. has acted as a speaker/lecturer for China Association for Alzheimer‘s Disease (CAAD), Taipei Medical University, Cleveland Clinic, Banner Health, AD/PD Congress, CTAD Congress Foundation of Learning; Health Society (Japan), INSPIRE Project U. Toulouse, Japan Society for Dementia Research, Korean Dementia Society, Merck Sharp & Dohme Corp., National Center for Geriatrics and Gerontology (NCGG); Japan, USC, and University of Wisconsin‐Madison. M.W. has served on advisory boards for ADNI, UCSF Committee for Human Research, ProMIS Neurosciences, Inc; Acumen Pharmaceutical SAB, and Duke University NC Registry for Brain Health SAB. M.W. holds leadership positions at UCSF Inclusion Diversity Equity & Awareness Committee and Diversity Task Force of the Alzheimer's Disease Neuroimaging Initiative (ADNI). M.W. holds stock options with Alzeca, ALZPath, Anven, and Alzheon, Inc. S.C.B. is an employee and shareholder of Eli Lilly and Co. Ltd. Y.Y.L. is the recipient of the National Health and Medical Research Council (NHMRC) Emerging Leadership Grant (GNT2009550). Y.Y.L. has received support for attending Alzheimer's Association as part of the Scientific Program Committee and serves as a section editor of *Alzheimer's & Dementia: Behavior & Socioeconomics of Aging*. The other authors declare no conflicts of interest. Author disclosures are available in the Supporting Information.

## CONSENT STATEMENT

All participants from the AIBL study provided written consent for publication of de‐identified data. Participants from the ADNI cohort signed written informed consent for participation in the ADNI study, approved by the Institutional Review Board at each participating center. All OASIS‐3 participants consented to the use of their data by the scientific community, and data sharing terms have been approved by the Washington University Human Research Protection Office.

## Supporting information



Supporting Information

Supporting Information

## References

[1] Baker JE , Lim YY , Pietrzak RH , et al. Cognitive impairment and decline in cognitively normal older adults with high amyloid‐β: a meta‐analysis. Alzheimers Dement (Amst). 2017;6:108‐121. 10.1016/j.dadm.2016.09.002 PMC5315443 28239636

[2] Tam A , Laurent C , Gauthier S , Dansereau C . Prediction of cognitive decline for enrichment of Alzheimer's disease clinical trials. J Prev Alzheimers Dis. 2022;9:400‐409. 10.14283/jpad.2022.49 35841241

[3] Jonaitis EM , Koscik RL , Clark LR , et al. Measuring longitudinal cognition: individual tests versus composites. Alzheimers Dement (Amst). 2019;11:74‐84. 10.1016/j.dadm.2018.11.006 PMC6816509 31673596

[4] Mormino EC , Papp KV . Amyloid accumulation and cognitive decline in clinically normal older individuals: implications for aging and early Alzheimer's disease. J Alzheimers Dis. 2018;64:S633. 10.3233/JAD-179928 PMC6387885 29782318

[5] Langbaum JB , Hendrix SB , Ayutyanont N , et al. An empirically derived composite cognitive test score with improved power to track and evaluate treatments for preclinical Alzheimer's disease. Alzheimers Dement. 2014;10:666‐674. 10.1016/j.jalz.2014.02.002 PMC4201904 24751827

[6] Lim YY , Snyder PJ , Pietrzak RH , et al. Sensitivity of composite scores to amyloid burden in preclinical Alzheimer's disease: introducing the Z‐scores of attention, verbal fluency, and episodic memory for nondemented older adults composite score. Alzheimers Dement (Amst). 2016;2:19‐26. 10.1016/j.dadm.2015.11.003 PMC4879646 27239532

[7] Malek‐Ahmadi M , Chen K , Perez SE , He A , Mufson EJ . Cognitive composite score association with Alzheimer's disease plaque and tangle pathology. Alzheimers Res Ther. 2018;10:90. 10.1186/s13195-018-0401-z PMC6134796 30205840

[8] Langbaum JB , Ellison NN , Caputo A , et al. The Alzheimer's prevention initiative composite cognitive test: a practical measure for tracking cognitive decline in preclinical Alzheimer's disease. Alzheimers Res Ther. 2020;12:66. 10.1186/s13195-020-00633-2 PMC7254761 32460855

[9] Liu S , Maruff P , Fedyashov V , Masters CL , Goudey B . A data‐driven cognitive composite sensitive to amyloid‐β for preclinical Alzheimer's disease. J Alzheimers Dis. 2024;101:889‐899. 10.3233/JAD-231319 39269831

[10] Donohue MC , Sperling RA , Salmon DP , et al. The preclinical Alzheimer cognitive composite: measuring amyloid‐related decline. JAMA Neurol. 2014;71:961‐970. 10.1001/jamaneurol.2014.803 PMC4439182 24886908

[11] Bransby L , Lim YY , Ames D , et al. Sensitivity of a preclinical Alzheimer's cognitive composite (PACC) to amyloid β load in preclinical Alzheimer's disease. J Clin Exp Neuropsychol. 2019;41:591‐600. 10.1080/13803395.2019.1593949 30924399

[12] Kansal A , Stern S , Keenan A . Simulation study on differences in Alzheimer disease (AD) cooperative study–preclinical Alzheimer cognitive composite (ADCS‐PACC) based on long‐term clinical outcomes (P5.193). Neurology. 2018;90: P5.193.

[13] Stricker NH , Twohy EL , Albertson SM , et al. Mayo‐PACC: a parsimonious preclinical Alzheimer's disease cognitive composite comprised of public‐domain measures to facilitate clinical translation. Alzheimers Dement. 2023;19:2575‐2584. 10.1002/alz.12895 PMC10272034 36565459

[14] Tallapragada B , Markovic SJ , Marston KJ , et al. Composite scores to detect and monitor cognitive dysfunction in preclinical and prodromal Alzheimer's disease: a narrative review. J Alzheimers Dis. 2026;111:489‐504. 10.1177/13872877261433044 41870260

[15] Rivera‐Fernández C , Custodio N , Soto‐Añari M . Neuropsychological profile in the preclinical stages of dementia: principal component analysis approach. Dement Neuropsychol. 2021;15:192‐199. 10.1590/1980-57642021dn15-020006 PMC8283881 34345360

[16] Ramanan S , Akarca D , Henderson SK , et al. The graded multidimensional geometry of phenotypic variation and progression in neurodegenerative syndromes. Brain. 2024;148:448‐466. 10.1093/brain/awae233 PMC11788217 39018014

[17] Hahn A , Kim YJ , Kim HJ , et al. The preclinical amyloid sensitive composite to determine subtle cognitive differences in preclinical Alzheimer's disease. Sci Rep. 2020;10:13583. 10.1038/s41598-020-70386-3 PMC7423599 32788669

[18] Petersen RC , Aisen PS , Beckett LA , et al. Alzheimer's disease neuroimaging initiative (ADNI) clinical characterization. Neurology. 2010;74:201‐209. 10.1212/WNL.0b013e3181cb3e25 PMC2809036 20042704

[19] Fowler C , Rainey‐Smith SR , Bird S , et al. Fifteen years of the Australian imaging, biomarkers and lifestyle (AIBL) study: progress and observations from 2,359 older adults spanning the spectrum from cognitive normality to Alzheimer's disease. J Alzheimers Dis Rep. 2021;5:443‐468. 10.3233/ADR-210005 PMC8293663 34368630

[20] Marcus DS , Fotenos AF , Csernansky JG , Morris JC , Buckner RL . Open access series of imaging studies: longitudinal MRI data in nondemented and demented older adults. J Cogn Neurosci. 2010;22:2677‐2684. 10.1162/jocn.2009.21407 PMC2895005 19929323

[21] Shishegar R , Doecke JD , Lim YY , et al. Harmonizing neuropsychological test data across prospective studies. Alzheimers Dement. 2026;22:e71186. 10.1002/alz.71186 PMC12906370 41690816

[22] Bourgeat P , Doré V , Burnham SC , et al. β‐amyloid PET harmonisation across longitudinal studies: application to AIBL, ADNI and OASIS3. NeuroImage. 2022;262:119527. 10.1016/j.neuroimage.2022.119527 PMC9550562 35917917

[23] Bourgeat P , Doré V , Rowe CC , et al. A universal neocortical mask for centiloid quantification. Alzheimers Dement. 2023;15:e12457. 10.1002/dad2.12457 PMC10363815 37492802

[24] Burnham SC , Cox T , Benzinger T , et al. When does Alzheimer's disease start? Robust estimates based on longitudinal Aβ‐amyloid‐PET in three large international cohorts. Available at SSRN: https://ssrn.com/abstract=4757682 or 10.2139/ssrn.4757682

[25] Shishegar R , Cox T , Rolls D , et al. Using imputation to provide harmonized longitudinal measures of cognition across AIBL and ADNI. Sci Rep. 2021;11:23788. 10.1038/s41598-021-02827-6 PMC8664816 34893624

[26] R Core Team . R: a language and environment for statistical computing. R foundation for statistical computing. 2019. https://www.R-project.org

[27] Revelle W . psych: Procedures for psychological, psychometric, and personality research. R package version 1.9. 12. Evanston (Northwestern University; 2019).

[28] Ramanan S , Roquet D , Goldberg Z‐L , et al. Establishing two principal dimensions of cognitive variation in logopenic progressive aphasia. Brain Commun. 2020;2:fcaa125. 10.1093/braincomms/fcaa125 PMC7750924 33376980

[29] Riordan H . Constructing composites to optimise cognitive outcomes. J Clin Stud. 2017;9:40‐45.

[30] Bates D , Mächler M , Bolker B , Walker S . Fitting linear mixed‐effects models using lme4. J Stat Softw. 2015;67:48.

[31] Kuznetsova A , Brockhoff PB , Christensen RHB . lmerTest package: tests in linear mixed effects models. J Stat Softw. 2017;82:26.

[32] Therneau TM , Lumley T . Package ‘survival’. R Top Doc. 2015;128(10):28–33. doi:10.32614/CRAN.package.survival

[33] Chen T , Guestrin C . XGBoost: a scalable tree boosting system. Proceedings of the 22nd ACM SIGKDD international conference on knowledge discovery and data mining. Association for Computing Machinery; 2016. 785‐794.

[34] Kuhn , et al. Tidymodels: a collection of packages for modeling and machine learning using tidyverse principles. 2020. https://www.tidymodels.org

[35] Pereira T , Lemos L , Cardoso S , et al. Predicting progression of mild cognitive impairment to dementia using neuropsychological data: a supervised learning approach using time windows. BMC Med Inform Decis Mak. 2017;17:110. 10.1186/s12911-017-0497-2 PMC5517828 28724366

[36] Kaiser HF , Rice J . Little Jiffy, mark Iv. Educ Psychol Meas. 1974;34:111‐117.

[37] Schielzeth H , Dingemanse NJ , Nakagawa S , et al. Robustness of linear mixed‐effects models to violations of distributional assumptions. Methods Ecol Evol. 2020;11:1141‐1152.

[38] van der Kall LM , Truong T , Burnham SC , et al. Association of β‐amyloid level, clinical progression, and longitudinal cognitive change in normal older individuals. Neurology. 2021;96:e662‐e670. 10.1212/WNL.0000000000011222 PMC7884996 33184233

[39] Harrington KD , Lim YY , Ellis KA , et al. The association of Aβ amyloid and composite cognitive measures in healthy older adults and MCI. Int Psychogeriatr. 2013;25:1667‐1677. 10.1017/S1041610213001087 23866942

[40] Schneider LS , Goldberg TE . Composite cognitive and functional measures for early stage Alzheimer's disease trials. Alzheimers Dement (Amst). 2020;12:e12017. 10.1002/dad2.12017 PMC7233425 32432155

[41] Mokkink LB , Prinsen CA , Bouter LM , Vet HC , Terwee CB . The COnsensus‐based standards for the selection of health measurement instruments (COSMIN) and how to select an outcome measurement instrument. Braz J Phys Ther. 2016;20:105‐113. 10.1590/bjpt-rbf.2014.0143 PMC4900032 26786084

[42] Clark LA , Watson D . Constructing validity: new developments in creating objective measuring instruments. Psychol Assess. 2019;31:1412‐1427. 10.1037/pas0000626 PMC6754793 30896212

[43] Thomas KR , Bangen KJ , Weigand AJ , et al. Cognitive heterogeneity and risk of progression in data‐driven subtle cognitive decline phenotypes. J Alzheimers Dis. 2022;90:323‐331. 10.3233/JAD-220684 PMC9661321 36120785

[44] Gibbons LE , Carle AC , Mackin RS , et al. A composite score for executive functioning, validated in Alzheimer's disease neuroimaging initiative (ADNI) participants with baseline mild cognitive impairment. Brain Imaging Behav. 2012;6:517‐527. 10.1007/s11682-012-9176-1 PMC3684181 22644789

[45] Dhareshwar S , Kane M , Lewis C , et al. Update on the current state of speech and language testing in Alzheimer's disease. J Alzheimers Dis. 2026;112:5‐19. 10.1177/13872877261446637 42081089

[46] Papp KV , Mormino EC , Amariglio RE , et al. Biomarker validation of a decline in semantic processing in preclinical Alzheimer's disease. Neuropsychology. 2016;30:624‐630. 10.1037/neu0000246 PMC4877295 26595826

[47] Murai SA , Mano T , Sanes JN , Watanabe T . Atypical intrinsic neural timescale in the left angular gyrus in Alzheimer's disease. Brain Commun. 2024;6:fcae199. 10.1093/braincomms/fcae199 PMC11227993 38993284

[48] Ossenkoppele R , Madison C , Oh H , Wirth M , van Berckel BN , Jagust WJ . Is verbal episodic memory in elderly with amyloid deposits preserved through altered neuronal function? Cereb Cortex. 2014;24:2210‐2218. 10.1093/cercor/bht076 PMC4089385 23537530

[49] Wagner J , Rusconi E . Causal involvement of the left angular gyrus in higher functions as revealed by transcranial magnetic stimulation: a systematic review. Brain Struct Funct. 2023;228:169‐196. 10.1007/s00429-022-02576-w PMC9813212 36260126

[50] Wada‐Isoe K , Kikuchi T , Umeda‐Kameyama Y , Mori T , Akishita M , Nakamura Y . Global clinical dementia rating score of 0.5 may not be an accurate criterion to identify individuals with mild cognitive impairment. J Alzheimers Dis Rep. 2019;3:233‐239. 10.3233/ADR-190126 PMC6839533 31754655

