# Supplementary material for: Data‑driven cognitive composites predict Aβ‑related decline and progression to MCI: the ADOPIC study
Source: Alzheimers Dement. 2026 Oct 11;22(10):e71917. doi: 10.1002/alz.71917 (PMC13655217; doi:10.1002/alz.71917)
Supplement: Supplementary file 2 [file ALZ-22-e71917-s001.docx]

**Supplementary Materials:** Data‑driven cognitive composites predict Aβ‑related decline and progression to MCI: The ADOPIC Study

Bhargav Tallapragada^1,2^, Brook Galna^1,3,4^, Rosita Shishegar^5,6^, Shaun J. Markovic^7^, Belinda M. Brown^1,8,9^, Vincent Dore^5^, Simon M. Laws ^10,11,12^, Tenielle Porter^10,11,12^; Samantha Burnham^5^, Yen Ying Lim^13^, Jason Hassenstab^14^, Christopher C. Rowe^15^, Liang Jin^16^, Jurgen Fripp^17^, James D. Doecke^10,17^, Duygu Tosun^18,19^, Michael Weiner^18,19^, Ralph N. Martins^9,^ ^20, 23^, John C. Morris^21^, Colin L. Masters^9,23^, Paul Maruff^16,22^, Hamid R. Sohrabi^1,2, 9*^, on behalf of ADOPIC Study.

# **Participant inclusion**

## **Figure 1.** CONSORT diagram detailing the process of data subset selection

# **Selecting the machine learning classification algorithm**

## **Figure 2.** Balanced accuracy plot comparing the model performance across the four ML models.

*Note*: Balanced accuracy is (mean sensitivity + mean specificity) / 2, and the error bars denote the balanced accuracy standard error calculated by the square root of (standard error of sensitivity + standard error of specificity) / 2. All composites are z-scores standardized to a mean of 0 and a SD of 1.

*Abbreviations*: XGB, Extreme gradient boost; SVM, support vector machine; RF, random forest; Log, Logistic regression; L&M, Learning & Memory; EF, Executive Function; A&P, Attention & Processing Speed; GC, General Cognition; ADCS-PACC; Alzheimer’s Disease Cooperative Study – Preclinical Alzheimer’s Cognitive Composite; MMSE, Mini Mental State Examination.

# **Overall probability of remaining cognitively unimpaired across the cohorts**

## **Figure 3.** Kaplan Meier Plot Denoting overall survival probability by cohort

*Note*: On the x-axis, ‘Years’ represents the time from baseline in years. Overall survival probability is survival probability expressed in percentage.

*Abbreviations***:** ADNI, Alzheimer’s disease Neuroimaging Initiative; AIBL, Australian Imaging, Biomarkers, and Lifestyle Flagship study of ageing; OASIS, the Open Access Series of Imaging Studies.

# **Preliminary linear mixed model results to guide data subset selection**

Linear mixed model (LMM) results presented in Figures 1-4 show each cognitive composite's fixed and random effects. Of note, Baseline Amyloid-beta (Aβ) status was not included in any of the models, as the data in the current analysis included baseline-only data; therefore, for a fair comparison, all four models are presented without baseline Aβ as a predictor. Examining the fixed, interaction, and random effects across the four figures S1, S2, S3, and S4 shows that the estimates of effects remained consistent across the four subsets. Notably, *APOE* ε4 carriage status and time interaction emerged significant (*p* < 0.05) across most models. The marginal and conditional R^2^ values were nearly identical across the subsets, and notably, the eight-year subset maintained 99.9% of participants (1,110 of 1,111) and 94.6% of observations (5,297 of 5,601) compared to the full dataset. As noted in the methods section of the manuscript, one of the cohorts, OASIS, did not have follow-up data beyond 8.35 years. Together the results from the models above and in consideration of the available follow-up data from OASIS, the eight years from baseline was selected as the final subset for the analysis in the current study.

## **Figure 4**. Linear mixed model estimates for memory across various subsets of data

*Note*: *APOE* ε4 (0), i.e., non-carriers and Sex (female) were the reference levels. σ² denotes the residual variance, and τ00 denotes the variance of the random intercepts. τ11 represents the variance of the random slopes. ϱ01 shows the correlation between random intercepts and random slopes. ICC represents the proportion of the total variance explained by the grouping structure. L&M composite is a z-score standardized to a mean of 0 and a SD of 1.

*Abbreviations*: L&M, Learning & Memory; apoe binary, Apolipoprotein ε4 allele carriage status (0 – non-carrier, 1 – carrier); YoE, Years of education; σ², sigma squared; τ00, tau-zero-zero; τ11, tau-one-one; ϱ01, rho-zero-one; ICC, Intraclass Correlation Coefficient; N, number of observations

## **Figure 5**. Linear mixed model estimates for executive function across various subsets of data

*Note*: *APOE* ε4 (0), i.e., non-carriers and Sex (female) were the reference levels. σ² denotes the residual variance, and τ00 denotes the variance of the random intercepts. τ11 represents the variance of the random slopes. ϱ01 shows the correlation between random intercepts and random slopes. ICC represents the proportion of the total variance explained by the grouping structure. EF composite is a z-score standardized to a mean of 0 and a SD of 1.

*Abbreviations*: EF, Executive Function; apoe binary, Apolipoprotein ε4 allele carriage status (0 – non-carrier, 1 – carrier); YoE, Years of education; σ², sigma squared; τ00, tau-zero-zero; τ11, tau-one-one; ϱ01, rho-zero-one; ICC, Intraclass Correlation Coefficient; N, number of observations

## **Figure 6**. Linear mixed model estimates for attention across various subsets of data

*Note*: *APOE* ε4 (0), i.e., non-carriers and Sex (female) were the reference levels. σ² denotes the residual variance, and τ00 denotes the variance of the random intercepts. τ11 represents the variance of the random slopes. ϱ01 shows the correlation between random intercepts and random slopes. ICC represents the proportion of the total variance explained by the grouping structure. A&P composite is a z-score standardized to a mean of 0 and a SD of 1.

*Abbreviations*: A&P, Attention and Processing speed; apoe binary, Apolipoprotein ε4 allele carriage status (0 – non-carrier, 1 – carrier); YoE, Years of education; σ², sigma squared; τ00, tau-zero-zero; τ11, tau-one-one; ϱ01, rho-zero-one; ICC, Intraclass Correlation Coefficient; N, number of observations

## **Figure 7**. Linear mixed model estimates for General Cognition across various subsets of data

*Note*: GC, General Cognition. *APOE* ε4 (0), i.e., non-carriers and Sex (female) were the reference levels. σ² denotes the residual variance, and τ00 denotes the variance of the random intercepts. τ11 represents the variance of the random slopes. ϱ01 shows the correlation between random intercepts and random slopes. ICC represents the proportion of the total variance explained by the grouping structure. GC composite is a z-score standardized to a mean of 0 and a SD of 1.

*Abbreviations*: APOE binary, Apolipoprotein ε4 allele carriage status (0 – non-carrier, 1 – carrier); YoE, Years of education; σ², sigma squared; τ00, tau-zero-zero; τ11, tau-one-one; ϱ01, rho-zero-one; ICC, Intraclass Correlation Coefficient; N, number of observations

# **Linear mixed model assumption diagnostic plots**

Diagnostic plots for all the final LMMs reported in the manuscript are presented in Supplementary Figures 8-11. Visual inspection of posterior predictive checks showed good alignment between observed and model-predicted distributions across all cognitive domains, indicating adequate overall model fit. Residual plots examining linearity and homogeneity of variance revealed minor deviations, with some heteroscedasticity, particularly at extreme fitted values, and an interesting striping pattern was observed in the Attention LMM. All models showed low multicollinearity with a variance inflation factor consistently below 2 for all predictors. Q-Q plots indicated deviations from normality in residual distributions, particularly in the tails. Random effects structures showed reasonable normality with some deviations at the extremes. While these minor violations were observed, linear mixed models are generally robust to minor assumption violations.

## **Figure 8**. Diagnostic plot for Learning & Memory(z) linear mixed model

## **Figure 9**. Diagnostic plot for Executive Function (z) linear mixed model

## **Figure 10**. Diagnostic plot for Attention & Processing Speed (z) linear mixed model

## **Figure 11**. Diagnostic plot for General Cognition (z) linear mixed model

# **Linear mixed model final model selection and model building**

Findings from Supplementary Tables 1 – 4 present the various model summary statistics used to select the final model. In both Learning & Memory and General Cognition LMMs, each incremental predictor resulted in a statistically significant (*p* < 0.05) improvement in model fit across the range of summary statistics. Similarly, the Executive Function LMMs showed significant improvements with all predictors except the main effect of *APOE* ε4 (*p* = 0.104), while its interaction with time remained significant (p = 0.005). For Attention & Processing Speed, both *APOE* ε4 × Time (*p* = 0.016) and baseline Aβ status × Time (*p* < .0001) interactions significantly improved model fit, but the Baseline Age × Time interaction did not (*p* = 0.285). The most substantial improvements in model fit were observed when adding the baseline Aβ status × Time interaction, particularly for General Cognition (χ² = 103.30) and Memory (χ² = 68.2), confirming the known findings on baseline amyloid pathology strongly influences longitudinal trajectories in these domains. These results support our final model selection, including all significant interaction terms for each final LMM reported in the manuscript.

## **Table 1**. Linear mixed model building and selection for Learning and Memory (z)

| Model | Fixed effects added | npar | AIC | BIC | Log-likelihood | Deviance | χ2 | Df | *p*-value |
| --- | --- | --- | --- | --- | --- | --- | --- | --- | --- |
| Base | Baseline age + Sex + Time + Education | 09 | 10, 698 | 10, 757 | -5340 | 10, 680 | - | - | - |
| + Aβ status | Baseline Aβ status | 10 | 10, 688 | 10, 754 | -5334 | 10, 668 | 11.3 | 1 | < 0. 001 |
| + *APOE* ε4 | *APOE* ε4 carriage status | 11 | 10, 680 | 10, 752 | -5329 | 10, 658 | 10.1 | 1 | < 0.001 |
| + *APOE* ε4 x time | *APOE* ε4 x time | 12 | 10, 667 | 10, 746 | -5321 | 10, 643 | 15.2 | 1 | < 0.001 |
| + Aβ status x time | Baseline Aβ status x time | 13 | 10, 601 | 10, 686 | -5287 | 10, 575 | 68.2 | 1 | < 0.001 |
| + Baseline age x time | Baseline age x time | 14 | 10, 572 | 10, 664 | -5272 | 10, 544 | 30.6 | 1 | < 0.001 |

*Note*: All models include random intercepts and slopes for participants. χ² values and *p*-values represent likelihood ratio tests comparing each model to the previous one. Age was centred at 72.6 years; Education was centred at 14.6 years. Female participants, Aβ – and *APOE* ε4 non-carriers were the reference levels.

*Abbreviations*: Aβ status, Amyloid beta status; *APOE* ε4, Apolipoprotein ε4 allele carriage status; AIC, Akaike Information Criteria; BIC, Bayesian Information Criteria; χ2, chi-square statistic; Df, degrees of freedom*.*

## **Table 2**. Linear mixed model building and selection for Executive Function (z)

| Model | Fixed effects added | npar | AIC | BIC | Log-likelihood | Deviance | χ2 | Df | *p*-value |
| --- | --- | --- | --- | --- | --- | --- | --- | --- | --- |
| Base | Baseline age + Sex + Time + Education | 09 | 10, 539 | 10, 598 | -5260 | 10, 521 | - | - | - |
| + Aβ status | Baseline Aβ status | 10 | 10, 528 | 10, 594 | -5254 | 10, 508 | 12.51 | 1 | < 0. 001 |
| + *APOE* ε4 | *APOE* ε4 carriage status | 11 | 10, 528 | 10, 600 | -5253 | 10, 506 | 2.65 | 1 | 0.103 |
| + *APOE* ε4 x time | *APOE* ε4 x time | 12 | 10, 522 | 10, 601 | -5249 | 10, 498 | 7.87 | 1 | 0.005 |
| + Aβ status x time | Baseline Aβ status x time | 13 | 10, 460 | 10, 545 | -5217 | 10, 434 | 64.10 | 1 | < 0.001 |
| + Baseline age x time | Baseline age x time | 14 | 10, 383 | 10, 475 | -5178 | 10, 355 | 78.47 | 1 | < 0.001 |

*Note*: All models include random intercepts and slopes for participants. χ² values and *p*-values represent likelihood ratio tests comparing each model to the previous one. Age was centred at 72.6 years; Education was centred at 14.6 years. Female participants, Aβ – and *APOE* ε4 non-carriers were the reference levels.

*Abbreviations*: Aβ status, Amyloid beta status; *APOE* ε4, Apolipoprotein ε4 allele carriage status; AIC, Akaike Information Criteria; BIC, Bayesian Information Criteria; χ2, chi-square statistic; Df, degrees of freedom.

## **Table 3**. Linear mixed model building and selection for Attention and Processing Speed (z)

| Model | Fixed effects added | npar | AIC | BIC | Log-likelihood | Deviance | χ2 | Df | *p*-value |
| --- | --- | --- | --- | --- | --- | --- | --- | --- | --- |
| Base | Baseline age + Sex + Time + Education | 07 | 7, 866 | 7, 912 | -3926 | 7, 852 | - | - | - |
| + Aβ status | Baseline Aβ status | 08 | 7, 865 | 7, 918 | -3924 | 7, 849 | 2.62 | 1 | 0.105 |
| + *APOE* ε4 | *APOE* ε4 carriage status | 09 | 7, 864 | 7, 923 | -3923 | 7, 846 | 2.69 | 1 | 0.101 |
| + *APOE* ε4 x time | *APOE* ε4 x time | 10 | 7, 860 | 7, 926 | -3920 | 7, 840 | 5.78 | 1 | 0.016 |
| + Aβ status x time | Baseline Aβ status x time | 11 | 7, 844 | 7, 916 | -3911 | 7, 822 | 18.62 | 1 | < 0.001 |
| + Baseline age x time | Baseline age x time | 12 | 7, 845 | 7, 924 | -3910 | 7, 821 | 1.14 | 1 | 0.285 |

*Note*: All models include random intercepts and slopes for participants. χ² values and *p*-values represent likelihood ratio tests comparing each model to the previous one. Age was centred at 72.6 years; Education was centred at 14.6 years. Female participants, Aβ – and *APOE* ε4 non-carriers were the reference levels.

*Abbreviations*: Aβ status, Amyloid beta status; *APOE* ε4, Apolipoprotein ε4 allele carriage status; AIC, Akaike Information Criteria; BIC, Bayesian Information Criteria; χ2, chi-square statistic; Df, degrees of freedom.

## **Table 4**. Linear mixed model building and selection for General Cognition (z)

| Model | Fixed effects added | npar | AIC | BIC | Log-likelihood | Deviance | χ2 | Df | *p*-value |
| --- | --- | --- | --- | --- | --- | --- | --- | --- | --- |
| Base | Baseline age + Sex + Time + Education | 09 | 5, 433 | 5, 492 | -2707 | 5, 415 | - | - | - |
| + Aβ status | Baseline Aβ status | 10 | 5, 422 | 5, 488 | -2701 | 5, 402 | 12.57 | 1 | < 0.001 |
| + *APOE* ε4 | *APOE* ε4 carriage status | 11 | 5, 414 | 5, 486 | -2696 | 5, 392 | 9.97 | 1 | < 0.001 |
| + *APOE* ε4 x time | *APOE* ε4 x time | 12 | 5, 396 | 5, 475 | -2686 | 5, 372 | 20.18 | 1 | < 0.001 |
| + Aβ status x time | Baseline Aβ status x time | 13 | 5, 295 | 5, 380 | -2634 | 5, 269 | 103.30 | 1 | < 0.001 |
| + Baseline age x time | Baseline age x time | 14 | 5, 239 | 5, 332 | -2606 | 5, 211 | 57.17 | 1 | < 0.001 |

*Note*: All models include random intercepts and slopes for participants. χ² values and *p*-values represent likelihood ratio tests comparing each model to the previous one. Age was centred at 72.6 years; Education was centred at 14.6 years. Female participants, Aβ – and *APOE* ε4 non-carriers were the reference levels.

*Abbreviations*: Aβ status, Amyloid beta status; *APOE* ε4, Apolipoprotein ε4 allele carriage status; AIC, Akaike Information Criteria; BIC, Bayesian Information Criteria; χ2, chi-square statistic; Df, degrees of freedom.

# **Effect size differences in longitudinal sensitivity of the composites comparing CUAβ- vs. CUAβ+ groups**

## **Figure 12.** Longitudinal sensitivity of cognitive composites in assessing AB-related cognitive decline (Jackknife comparisons)

*Abbreviation*: MMSE, Mini Mental State Examination; Att’n & Proc. Speed, Attention and Processing Speed Composite; ADCS-PACC, Alzheimer’s Disease Cooperative Study – Preclinical Alzheimer’s Cognitive Composite. All composites are z-scores standardized to a mean of 0 and SD of 1.

*Note*: Cohen's *d* values with 95% CIs for the difference in annual slopes between CUAβ+ and CUAβ- groups, derived from linear mixed models controlling for age, sex, education, and *APOE* ε4 status. Negative values indicate steeper decline in CU Aβ+ compared to CU Aβ-. The vertical dashed line represents no group difference (*d* = 0). Asterisks indicate statistical significance (*p* < 0.001).

# **Cox regression model building**

The final model for Cox proportional hazards was selected using a stepwise forward selection process based on the Akaike Information Criterion (AIC). The base model included demographics only, i.e., baseline age, sex, and education as predictors. Cognitive composite scores (domain-specific), baseline Aβ status (positive or negative) and *APOE* ε4 allele carriage (non-carrier or carrier) were incrementally added as predictors. Table S1 presents the step-by-step selection process. Learning & Memory was selected first, as it showed the greatest improvement in the model fit (AIC reduced from 3911 to 3794). Baseline Aβ status was then added to the model (reduced the AIC to 3755), followed by Attention & Processing Speed (AIC: 3748) and Executive Function (AIC: 3747). The *APOE* ε4 carriage status was added in the final step. However, the AIC value remained the same (3747). Based on these findings, the model containing all available predictors was selected as the final model, i.e., demographic factors (Sex, Age, Education), Baseline Aβ status, *APOE* ε4 carriage status, and domain-specific cognitive measures. While some variables (*APOE* ε4 carriage status and Executive Function) did not significantly reduce AIC in later steps, they were retained based on the selection algorithm's criteria and theoretical relevance. The same model structure was then followed for General Cognition and ADCS-PACC.

## **Table 5**. Forward stepwise selection AIC values for incremental Cox regression models

| **Step** | **Variables included** | **Variables added** | **AIC** | **ΔAIC** |
| --- | --- | --- | --- | --- |
| 0 | Baseline age, sex, and education | - | 3911 | - |
| 1 | Baseline age, sex, education, and Learning & memory | Learning & Memory | 3794 | -117 |
| 2 | Baseline age, sex, education, Learning & Memory, and baseline Aβ status | Baseline Aβ status | 3755 | -39 |
| 3 | Baseline age, sex, education, Learning & Memory, baseline Aβ status, and Attention & Processing Speed | Attention & Processing Speed | 3748 | -7 |
| 4 | Baseline age, sex, education, Learning & Memory, baseline Aβ status, Attention & Processing Speed, and Executive Function | EXECUTIVE FUNCTION | 3747 | -1 |
| 5 | Baseline age, sex, education, Learning & Memory, baseline Aβ status, Attention & Processing Speed, Executive Function, and *APOE* ε4 carriage status | *APOE* ε4 carriage status | 3747 | 0 |

*Note*: Aβ positivity was defined as Aβ-PET centiloid score > 20. All cognitive composite scores are standardised z-scores. ΔAIC denoted the reduction in AIC value when a predictor was added. All composites are z-scores standardised to a mean of 0 and SD of 1.

*Abbreviations*: Aβ status, Amyloid-beta status (positive or negative); *APOE* ε4 carriage status, Apolipoprotein ε4 allele carriage status; ΔAIC, delta AIC.

# **XGBoost Model Performance Estimates**

## **Table 6**. Bootstrapping metrics of the XGBoost model across different predictor combinations of the test set

| Model | **ROC-AUC** | | **Sensitivity** | | **Specificity** | | **PPV** | | **NPV** | |
| --- | --- | --- | --- | --- | --- | --- | --- | --- | --- | --- |
|  | Estimate  (SE) | 95% CI | Estimate  (SE) | 95% CI | Estimate  (SE) | 95% CI | Estimate  (SE) | 95% CI | Estimate  (SE) | 95% CI |
| Learning & Memory(z) | 0.705  (0.001) | (0.703 to 0.706) | 0.621  (0.002) | (0.618 to 0.625) | 0.692  (0.001) | (0.69 to 0.694) | 0.296  (0.001) | (0.294 to 0.297) | 0.897  (0) | (0.897 to 0.899) |
| Executive Function (z) | 0.663  (0.001) | (0.662 to 0.665) | 0.565  (0.002) | (0.561 to 0.568) | 0.686  (0.001) | (0.683 to 0.688) | 0.272  (0.001) | (0.271 to 0.274) | 0.884  (0) | (0.883 to 0.885) |
| Attention & Processing Speed(z) | 0.667  (0.001) | (0.666 to 0.669) | 0.578  (0.002) | (0.574 to 0.582) | 0.656  (0.001) | (0.654 to 0.659) | 0.259  (0.001) | (0.258 to 0.261) | 0.882  (0) | (0.882 to 0.883) |
| General Cognition(z) | 0.707  (0.001) | (0.706 to 0.709) | 0.637  (0.002) | (0.633 to 0.64) | 0.684  (0.001) | (0.682 to 0.686) | 0.296  (0.001) | (0.294 to 0.297) | 0.901  (0) | (0.9 to 0.902) |
| ADCS-PACC(z) | 0.701  (0.001) | (0.700 to 0.703) | 0.572  (0.002) | (0.569 to 0.576) | 0.72  (0.001) | (0.718 to 0.722) | 0.299  (0.001) | (0.297 to 0.301) | 0.89  (0) | (0.89 to 0.891) |
| MMSE(z) | 0.665  (0.001) | (0.664 to 0.667) | 0.571  (0.002) | (0.568 to 0.575) | 0.657  (0.001) | (0.654 to 0.66) | 0.258  (0.001) | (0.256 to 0.259) | 0.881  (0) | (0.880 to 0.882) |

*Note*: Each model included a baseline cognitive composite score, baseline Aβ-PET centiloid score, *APOE* ε4 carriage status, baseline age, sex, and education. The outcome variable was progression status at 8.35 years (CN or MCI). All composites are z-scores standardized to a mean of 0 and SD of 1.

*Abbreviations*: Learning & Memory, Learning and Memory composite score; Executive Function Executive Function composite score; Attention & Processing Speed, Attention and processing speed composite score; ADCS-PACC, Alzheimer's Disease Cooperative Study Preclinical Alzheimer's Cognitive Composite; MMSE, Mini Mental State Examination; ROC-AUC, Receiver Operating Characteristic of the Area Under the Curve; PPV, Positive Predictive Value; NPV, Negative Predictive Value; S.E, Standard error; 95% CI, 95% Confidence Interval.

**Table 7**. Baseline group differences between CU Aβ- and Aβ+ on ADOPIC cognitive tests

| **Test** | **F** | **df** | **Aβ difference (z)** | ***p* (unadj.)** | ***p* (FDR)** |
| --- | --- | --- | --- | --- | --- |
| Trail Making Test-B (z) | **9.14** | **1** | **-0.165** | **0.003** | **0.041** |
| Mini-Mental State Examination (z) | 6.06 | 1 | -0.136 | 0.014 | 0.074 |
| Buschke Selective Reminding Test (free recall) (z) | 6.43 | 1 | -0.134 | 0.011 | 0.074 |
| Digit symbol coding (z) | 4.85 | 1 | -0.110 | 0.028 | 0.089 |
| Logical memory II (delayed recall) (z) | 5.02 | 1 | -0.118 | 0.025 | 0.089 |
| Digit span forward (z) | 4.41 | 1 | 0.112 | 0.036 | 0.095 |
| Rey auditory verbal learning test – recognition (z) | 3.98 | 1 | -0.109 | 0.046 | 0.104 |
| California Verbal Learning Test, second edition - delayed recall (z) | 3.78 | 1 | -0.104 | 0.052 | 0.104 |
| Rey Auditory Verbal Learning Test – delayed recall (z) | 3.16 | 1 | -0.096 | 0.076 | 0.135 |
| Verbal fluency switching (fruit and furniture) (z) | 2.72 | 1 | -0.086 | 0.099 | 0.158 |
| Rey Auditory Verbal Learning Test – total trials (z) | 2.14 | 1 | -0.076 | 0.144 | 0.209 |
| California Verbal Learning Test, Second Edition – total trials (z) | 1.32 | 1 | -0.061 | 0.251 | 0.334 |
| California verbal learning test, second edition – recognition (z) | 0.83 | 1 | -0.051 | 0.362 | 0.446 |
| Category fluency animals (total score) (z) | 0.40 | 1 | 0.033 | 0.525 | 0.601 |
| Boston Naming Test (no cue recall) (z) | 0.21 | 1 | -0.026 | 0.647 | 0.690 |
| Digit Span backward (z) | 0.07 | 1 | -0.015 | 0.777 | 0.777 |

*Note*: Baseline differences between CU Aβ- and Aβ+ estimated using GLM Type III SS, adjusted for baseline age, sex, education, and *APOE* ε4 carriage. All test scores were z-scores standardized to the CU Aβ- baseline group. The Aβ difference is expressed in standard-deviation units; a negative value indicates lower performance in the Aβ+ group. *p*-values were corrected for multiple comparisons across the 16 tests using the Benjamini-Hochberg false discovery rate (FDR). The analysis was conducted on *N* = 1,742 (participant with complete *APOE* and education) data. After correction, only Trail Making Test-B differed significantly between groups (*p*_FDR = 0.04).

*Abbreviations*: CU, Cognitively Unimpaired; Aβ, amyloid-beta; *APOE* ε4, Apolipoprotein ε4 allele; SS, Sum of Squares; FDR, false discovery rate; df, degrees of freedom.
